# Supplementary material for: Shearo-caloric effect enhances elastocaloric responses in polymer composites for solid-state cooling
Source: Nat Commun. 2024 Aug 3;15:6567. doi: 10.1038/s41467-024-50870-4 (PMC11297307; doi:10.1038/s41467-024-50870-4)
Supplement: Supplementary file 1 — Supplementary Information [file 41467_2024_50870_MOESM1_ESM.pdf]

Supplementary Information for

Shearo-caloric effect enhances elastocaloric responses in polymer composites for solid-state cooling

Shixian Zhang[1,2,6], Yuheng Fu[1,6], Xinxing Nie[1], Chenjian Li[1], Youshuang Zhou[3], Yaqi Wang[1], Juan Yi[1,4], Wenlai Xia[1], Yiheng Song[1], Qi Li[4], Chuanxi Xiong[1,7,*], Suxin Qian[5,*], Quanling Yang[1,*] and Qing Wang[2,7,*]

[1] State Key Laboratory of Silicate Materials for Architectures, and School of Materials Science and Engineering, Wuhan University of Technology, Wuhan 430070, China. [2] Department of Materials Science and Engineering, The Pennsylvania State University, University Park, PA, USA. [3] School of Materials Science and Engineering, Hubei University, Wuhan 430062, China. [4] Department of Electrical Engineering, Tsinghua University, Beijing100084, China. [5] Department of Refrigeration and Cryogenic Engineering, School of Energy and Power Engineering, Xi’an Jiaotong University, Xi’an, Shaanxi 710049, China. [6] These authors contributed equally: Shixian Zhang, Yuheng Fu. [7] These authors jointly supervised this work: Chuanxi Xiong, Qing Wang.

* Corresponding authors’ email: cxiong@whut.edu.cn (Chuanxi Xiong[1,*]); qiansuxin@xjtu.edu.cn (Suxin Qian[5,*]); yangql@whut.edu.cn (Quanling Yang[1,*]); wang@matse.psu.edu (Qing Wang[2,*]).

This Supplementary Information file includes:

Supplementary Methods

Supplementary Discussions 1 to 5

Supplementary Figs. 1 to 21

Supplementary Tables 1 to 5

Supplementary References 1 to 34

Supplementary Methods

Materials and preparation.

Three kinds of triblock poly(styrene-b-ethylene-co-butylene-b-styrene) (SEBS) thermoplastic elastomers, with polystyrene (PS) hard blocks surrounding a poly(ethylene-co-butylene) (PEB) central soft block, used in this study were provided by KRATON Polymers Co., Ltd. (G1650MV, http://www.kraton-polymers.cn/), TSRC Industries Co., Ltd. (6159, https://www.tsrc.com.tw/), and Northwest Rubber & Plastics Research & Design Institute Co., Ltd., China (as-40, http://www.xbxj.chemchina.com/xbxjy/index.htm). Graphene nanoplatelets (GNS) with thickness of 4-20 nm and size of 5-10 μm were provided by Chengdu Chemicals Co., Ltd, Chinese Academy of Science. Multi-walled carbon nanotubes (MCNT) with outside diameter of 5-15 nm and length of 10-30 μm were provided by Chengdu Chemicals Co., Ltd, Chinese Academy of Science. Conductive carbon black with diameter of about 50 nm was provided by Anhui Science Technology Co., Ltd., China. Methylbenzene was an analytical reagent obtained from Aladdin Bio-Chem Technology Co., Ltd. (Shanghai, China).

In a typical process, different amounts of nanofillers (GNS, MCNT, and CB) were dispersed into methylbenzene under one hour's sonification to prepare the nanofiller solutions. Then, commercial SEBS were dissolved in methylbenzene by magnetic stirring for two hours at room temperature to prepare the matrix solutions with a solid content of 15 wt%. After the matrix solutions were stirred to clear, the nanofiller solutions were proportionally added to the matrix solution and stirred for three days to prepare the nanofillers/SEBS slurries.[1] The as-prepared slurries were transferred to glass culture dishes and dried at 60 ^{o}C for two days in a vacuum oven to remove the trace methylbenzene. Finally, the nanofillers/SEBS composites sample sheets (0.5-1 mm thickness) were obtained. After that, the thermalgravimetric analysis (TGA, NETZSCH STA 2500) was

performed to test the thermogravimetric curves and derivative thermogravimetric (DTG) curves of the GNS/HHs (1 mm thickness). The results show that there is no weight loss related to solvent volatilization before the decomposition temperature of the matrix, which confirmed that there were no solvent residues in the sample sheets (details in Supplementary Fig. 4). Test specimens for two-dimensional wide-angle X-ray diffraction (2D WAXD), dynamic mechanical measurement (DMA) and mechanical characterization (ASTM D4482, active area is about 25 mm in length, 4 mm in width and 1 mm in thickness) and *e*-CE measurement (active area is about 30 mm in length, 20 mm in width and 0.5 mm in thickness) were die cut from the same molded composite sample sheet. Film samples with a thickness of about 10 μm were prepared by solvent-casting of 25 g L^{-1} nanofillers/SEBS slurries onto cleaned glass slides and used for the atomic force microscopy (AFM) test after drying at room temperature for seven days.[2, 3] All samples were kept at the required ambient temperature for ten minutes before testing. The fabrication, denotation, and labeling of nanofillers/SEBS composites and the mass fraction of nanofillers are shown in Supplementary Table 1.

The measuring device and the test methods of the *s*-CE enhanced *e*-CE ΔT_{adi}.

The ΔT_{adi} which contributed from both *s*-CE and *e*-CE in SEBS composites were directly measured by using a custom instrument, including a stretching device and temperature measuring device.[4, 5] The stretching device comprised a precision ball screw and a linear guide rail, driven by an AC servo motor and controller (ECMA, Delta Electronics Co., LTD). Two ends of the test specimen were mounted respectively on the endplate and the slide table of guide rail by jaw clamps. The servo motor drives the slide table to do precision repositioning movement on the linear guide rail, which causes the test specimen to undergo a controlled uniaxial strain. During the recovery process, the sider recovers its original position, and the specimen contracts spontaneously. Sample strain is

derived from the slider displacement. The ΔT_{adi} associated with the *e*-CEs in the SEBS samples was induced in an open indoor environment, heat sink and heat source involved in the heat exchange process were ambient air in this paper.

Direct measurement of ΔT_{adi} was performed by measuring the in-suit surface temperature variation of the deforming test specimen. The surface temperature of the deformed test specimen was measured by an on-line infrared thermometer (ABSD-01A, Aobosaide Automation Technology Co., LTD; BRW600-406, Hunan Firstrate Sencer Co., Ltd) and visualized by an infrared thermal imager (T890-2, Testo SE & Co. KGaA). The on-line infrared thermometer and the infrared thermal imager were fixed 10 cm above the plane of the test sample. The infrared emissivity of the infrared thermometer and the infrared thermal image was adjusted to that of the test sample (0.91), which was controlled by the built-in software of the thermal imager. The infrared emissivity of the test samples was determined by using a dual-band emissivity meter (IR-2, Shanghai Chengbo Photoelectric Technology Co., Ltd.) in 8-14 μm bands at room-temperature. Temperature information detected by an infrared thermometer was recorded at a frame rate of 9 s^{-1}, and it was averaged on a flat area of ~ 0.5 cm^2.

Considering that the loading of carbon fillers may affect the thermal conductivity (see Supplementary Table 5) of the *e*-CE samples and further lead to the differences in the adiabatic strain rates of *e*-CE samples, direct measurement of the surface temperature changes ($|\Delta T|$) of HH, 1GNS/HH, and 8GNS/HH were performed in an open indoor environment at different strain rates. As shown in Supplementary Fig. 1, the tested $|\Delta T|$s of all samples increase with the increase of strain rates when the strain rates are less than 5 s^{-1}. This is caused by the convection and radiation heat transfer between the test sample and the ambient environment at low strain rates. When the

strain rates are greater than 5 s^{-1}, the $|\Delta T|$ values gradually become constant. This means that the heat transfer duration was shortened at higher strain rates, and a strain rate higher than 5 s^{-1} in an open indoor environment is equivalent to the adiabatic test condition. Since the adiabatic strain rate was estimated to be about 5 s^{-1}, the examination condition with a strain rate of 15 s^{-1}, which was far higher than the adiabatic strain rate, in an open indoor environment was selected to quantify ΔT_{adi}.[4] It is worth noting that the stretching and recovery process of the test specimen and the refreshing process of the infrared thermometer still take time, and heat exchange will occur between the test specimen and the environment during this time interval. This leads to the reported ΔT_{adi} value in this paper being an approximate value of the theoretical adiabatic condition.

The CE activity in our work was only realized through the transition of chain conformations (entropy elasticity), whose input work path mainly depends on elongation. This means that the same sample, driven by different strains, has different values of CE. The highest CEs ΔT_{adi} of the same test specimen was determined under its ultimate strain (ε_u). In most studies, the ε_u is defined as the former strain before the fracture strain of a sample (detail of the fracture strains refers to the stress-strain curves in Supplementary Fig. 7).

Materials characterization.

The molecular properties of these SEBS were assessed by gel permeation chromatography (GPC, PL-GPC50, Agilent technologies, CA, USA). The GPC measurements were carried out at 40 °C using THF as the eluent at a flow rate of 1 mL min^{-1}. The chemical structures of SEBS samples were analyzed by ^1H-nuclear magnetic resonance (^1H-NMR) spectroscopy (Bruker, 400 MHz). TGA was performed with a heating rate of 10 K min^{-1} in the temperature range of 298–1273 K under the nitrogen atmosphere. Transmission electron microscopy (TEM) images were obtained

on FEI Tecnai G2 F30 with an accelerating voltage of 200 kV. The samples were ultramicrotomed at −130 °C to a section with a thickness of about 70 nm. The structure evolution of SEBS samples was in situ monitored by two-dimensional wide-angle X-ray diffraction (2D WAXD), which was performed using Genix 3D X beamline with wavelength λ = 1.54 Å at Xeuss 2.0 (Xenocs). The sample-to-detector distances for WAXD were set to be 151.7 mm. The value of the scattering wave vector magnitude is given by $q = \frac{4\pi \sin\theta}{\lambda}$ where 2θ is the scattering angle. The 2D WAXS measurements were taken immediately after the sample reached the predetermined strain. The exposure duration was set as 120 s for each picture. Mechanical tests were run in an electromechanical universal testing machine (E44.104, MTS systems Co., LTD) equipped with a BSA-XS-50kg force transducer and a CEC1200 temperature testing chamber. Dynamic mechanical properties of SEBS samples were carried out on a dynamic mechanical analyzer (DMA, PE-DMA8000) at 1 Hz with a heating rate of 2 K min^{-1} in the temperature range of 173–443 K. The specific heat capacity (c_p) was confirmed by the standard direct heat capacity measurement by using a differential scanning calorimetry (DSC, TA-DSC2500) instrument with a heating rate of 10 K min^{-1} in the temperature range of 203–443 K. Before testing the specific heat capacity, sapphire standard sample was used as the reference material for the Tzero and Direct heat capacity calibration of TA-DSC2500. Both calibration processes were performed with a heating rate of 10 K min^{-1} in the temperature range of 183–573 K. As the samples we used in this work did not involve a phase change such as strain-induced crystallization during the deformation process, the strain-dependence of c_p was ignored. All composite samples were placed in liquid nitrogen overnight, and the cross-sections of those after brittle fracture were used to investigate the morphology and dispersion of nanofillers by field-emission scanning electron microscope (FE-SEM, Hitachi SU8010). Atomic force microscopy (AFM, Bruker Dimension icon) was employed for imaging the morphology and phase of the composite samples. All experiments were carried

out with the same AFM probe under ambient conditions (temperature of 25℃, relative humidity of 25%). The morphological analyses and phase images were performed under ambient conditions using tapping mode. All quantitative measurements were carried out using a standard probe (0.01-0.025 Ohm-cm Antimony (n) doped Si, RTESP-150, Bruker) with a cantilever of 125 μm, resonant frequency of 150 kHz and spring constant k of 6 N m^{-1}. Thermal conductivity was performed by the hot disk thermal analyzer (TPS2500S, Hot Disk Instrument) by using the transient plate heat source method (ISO22007-2).

Model construction and simulation.

Model construction and simulation were performed by the Materials Studio Package and the Martini forcefield.[6] Supplementary Fig. 8 shows the chemical structure and the coarse-grained (CG) model of the SEBS and GNS. Each coordinate of the CG beads is the center of mass of the associated segment. According to the calculation results of ^{1}H-NMR, the Et/C value is 11% and the mass ratio of the PS hard block is 29 wt%. Therefore, each CG SEBS contains 100 butyl (B) beads, 100 ethyl (E) beads, and 62 styrene (S, 31 on each side of the PEB block) beads. This CG model of the SEBS with a molecular weight of 2.1×10^{4} g mol^{-1} can well represent the real SEBS chain.[7] The CG mapping of E and S beads follows our previous work and Xia et al, respectively.[5, 8] Then GNS was coarse grained by using the phenyl (P) rings as the subunit.

Periodic microstructures of pure SEBS and 1GNS/HH were constructed by using cubic boxes with edges up to 26 nm followed by geometry optimization with a convergence threshold for the specified maximum energy change of 10^{-3} kcal mol^{-1} and a convergence threshold for the specified maximum force of 0.5 kcal mol^{-1} $Å^{-1}$. After that, a Mesocite Dynamics with a temperature was employed by using an NPT ensemble to make these microstructures reach a steady state at 298.15

K.[9] Nosé method was adopted to produce a reliable thermostat where the *Q* ratio was 1.[10] Then a Mesocite Dynamics with uniaxial stress of 100 MPa was executed by using the Souza-Martins method.[11] The whole simulation time was 4000 ps, resulting in a strain of 6 for both boxes. The coordinates of the first and last two beads of each molecular chain were derived from the result file, and the linear distance between the two coordinates was calculated as the end-to-end distance.

Supplementary Discussions

Supplementary Discussion 1. Derivation of the energy conversion relationship between CE and conformational change during the CE cycle.

According to the traditional entropic elasticity theory, the internal energy of the elastomer system is constant in the process of the *e*-CE cycle, and the contribution of latent heat (corresponding to the first-order phase transition) is ignored. During the adiabatic stretching process (P1 to P2), the applied stress simply changes the conformation (ΔS_λ) of the elastomer molecular chain from the original crimp state to the extended state, so that external work ($\mathrm{d}W = f\mathrm{d}l$) can convert into sensible heat, i.e, $\mathrm{d}W = -T\mathrm{d}S_\lambda > 0$ ($\mathrm{d}W < 0$, when the system is doing work). Therefore, ΔT_{adi} during P1 to P2 can be deduced from the change of conformational entropy:

$$\Delta S_\lambda = -mc_{\mathrm{p}}\ln\frac{T_{\mathrm{h}}}{T_{\mathrm{a}}} \qquad \text{Supplementary Equation (1)}$$

where, *m* is the sample mass; c_{p} is the specific heat. When the molecular chains are Gaussian chains, and the motion of crosslinking points conforms to affine deformation, the conformational entropy change ΔS_λ can be further quantified by the statistical theory of rubber elasticity[12, 13]:

$$\Delta S_\lambda = -\frac{1}{2}Nk\left(\lambda^2 + \frac{2}{\lambda} - 3\right) \qquad \text{Supplementary Equation (2)}$$

where, N is the number of network chains, k is the Boltzmann's constant, λ is the principal elongation ratio of the elastic chain.[4] Based on Supplementary Equations (1) and (2), ΔT_{adi} is obtained as follows:

$$\Delta T_{\mathrm{adi}} \approx \frac{1}{2} NkT_{\mathrm{a}} \left(\lambda^2 + \frac{2}{\lambda} - 3\right)\left(\frac{1}{mc_{\mathrm{p}}}\right) \qquad \text{Supplementary Equation (3)}$$

During the heat transfer process with the heat sink (P2-P3), the applied strain remains constant, and the system reduces its temperature by releasing sensible heat until the thermal equilibrium is reached. Therefore, the change of thermal entropy per unit mass during the heat exchange process is as follows:

$$\Delta S_{\mathrm{iso}} = \frac{1}{m}\int_{T_{\mathrm{h}}}^{T_{\mathrm{a}}} \frac{1}{T} \mathrm{d}Q = \int_{T_{\mathrm{h}}}^{T_{\mathrm{a}}} \frac{c_{\mathrm{p}}}{T} \mathrm{d}T \qquad \text{Supplementary Equation (4)}$$

ΔS_{iso} is equal to ΔS_{λ} when the influence factors independent of entropy elasticity, such as internal friction, are ignored. Accordingly, during the retraction process of the deformed elastomer (P3-P4). The system works to reduce its temperature. ΔS_{λ} and ΔT_{adi} on the cooling process can be deduced as follows:

$$\Delta S_{\lambda} = \frac{1}{2} Nk \left(\lambda^2 + \frac{2}{\lambda} - 3\right) \qquad \text{Supplementary Equation (5)}$$

$$\Delta T_{\mathrm{adi}} = -\frac{1}{2} NkT_{\mathrm{a}} \left(\lambda^2 + \frac{2}{\lambda} - 3\right)\left(\frac{1}{mc_{\mathrm{p}}}\right) \qquad \text{Supplementary Equation (6)}$$

During the heat transfer process with heat source (P4-P1), the sample absorbs heat from the environment and finally returns to the initial state. The change of thermal entropy per unit mass during heat exchange process is as follow:

$$\Delta S_{\mathrm{iso}} = \frac{1}{m}\int_{T_4}^{T_{\mathrm{a}}} \frac{1}{T} \mathrm{d}Q = \int_{T_{\mathrm{c}}}^{T_{\mathrm{a}}} \frac{c_{\mathrm{p}}}{T} \mathrm{d}T \qquad \text{Supplementary Equation (7)}$$

In the composite system at macroscopic uniaxial deformation, the introduction of nanofillers can generate additional cross-linking points within unit mass via bonding with the interlayer molecular chains. The stress transfer in the composite network results in the relative dislocations between nanofillers, so that the molecular chains bonded between the nanofillers (shear unit) can be considered as additional effective elastic chains and can undergo the conformational changes driven by the shearing process of the nanofillers. Assuming that the total number of effective network chains of the composite system is $N = N_e + N_s$, where N_e is the effective number of network chains of the original matrix, and N_s is the added value of the effective network chains introduced from the slip of the nanofillers. ΔT_{adi} in Supplementary Equation (3) is obtained as follows:

$$\Delta T_{adi} \approx \frac{1}{2}(N_e + N_s)kT_a\left(\lambda^2 + \frac{2}{\lambda} - 3\right)\left(\frac{1}{mc_p}\right) \quad \text{Supplementary Equation (8)}$$

Considering molecular dynamics, the mean square end-to-end distance $\overline{h^2}$ is obtained as follows:

$$\overline{h^2} = \frac{N_e}{N}\frac{\sum_{i=1}^{N_e} h_i^2}{N_e} + \frac{N_s}{N}\frac{\sum_{i=N_e+1}^{N} h_i^2}{N_s} \quad \text{Supplementary Equation (9)}$$

where h_i is the end-to-end distance of a molecular chain. Based on Supplementary Equation (9), considering the four models of HH and 1GNS/HH shown in Fig. 2m, we have the following equations:

$$\overline{h_I^2} = \overline{h_{II}^2} = \frac{\sum_{i=1}^{N} h_i^2}{N} \quad \text{Supplementary Equation (10)}$$

$$\overline{h_{II}^{'2}} = \frac{N_e}{N}\frac{\sum_{i=1}^{N_e} h_i^{'2}}{N_e} + \frac{N_s}{N}\frac{\sum_{i=N_e+1}^{N} h_i^{'2}}{N_s} \quad \text{Supplementary Equation (11)}$$

$$\overline{h_I^{'2}} = \frac{N_e}{N}\frac{\sum_{i=1}^{N_e} h_i^{'2}}{N_e} + \frac{N_s}{N}\frac{\sum_{i=N_e+1}^{N} h_i^2}{N_s} \quad \text{Supplementary Equation (12)}$$

where, $\overline{h_{\mathrm{I}}^2}$ is the mean square end-to-end distance of the original matrix (HH) at the initial state, $\overline{h_{\mathrm{II}}^2}$ is the mean square end-to-end distance of the composite (1GNS/HH) at the initial state, $\overline{h_{\mathrm{I}}^{'2}}$ is the mean square end-to-end distance of the original matrix (HH) at elongation state, $\overline{h_{\mathrm{II}}^{'2}}$ is the mean square end-to-end distance of the composite (1GNS/HH) at elongation state, h_{i} is the end-to-end distance of a molecular chain at the initial state, and $h_{\mathrm{i}}^{'}$ is the end-to-end distance of a molecular chain at elongation state. Under the assumption of affine deformation, simultaneous Supplementary Equation (10)-(12), we have that:

$$\overline{h_{\mathrm{I}}^{'2}} = \frac{N_{\mathrm{e}}}{N}\overline{h_{\mathrm{II}}^{'2}} + \frac{N_{\mathrm{s}}}{N}\overline{h_{\mathrm{I}}^2} \qquad \text{Supplementary Equation (13)}$$

The statistical results in Fig. 2m indicated that $\overline{h_{\mathrm{I}}^2} \approx 4054$ Å^2, $\overline{h_{\mathrm{II}}^{'2}} \approx 91474$ Å^2 and $\overline{h_{\mathrm{I}}^{'2}} \approx 75707$ Å^2. Therefore, for 1GNS/HH system, $\frac{N_{\mathrm{s}}}{N_{\mathrm{e}}} \approx$ 22%, $\frac{N_{\mathrm{s}}}{N} \approx$ 18% and $\frac{N_{\mathrm{e}}}{N} \approx$ 82%. This indicates that for 1GNS/HH at a strain level of 6, the shear effect of GNSs on the interlayer molecular chains increases the number of effective network chains in unit mass ($\frac{N}{m}$) of the system, and thus further increases the ΔS_{λ} and the associated ΔT_{adi} of the system during deformation. The *s*-CE introduced from the dislocations of the nanofillers and the *e*-CE from the original matrix contribute 18% and 82% to the entire CE of 1GNS/HH under uniaxial tensile, respectively. Theoretically, the *s*-CE quantitatively increases the *e*-CE of the original matrix by about 22%. It can be considered that the ΔT_{adi} of HH is mainly attributed to the *e*-CE generated by the original network chains of the matrix, and the ΔT_{adi} of 1GNS/HH is attributed to the joint contribution of both original *e*-CE and the additional *s*-CE. The difference value between these two ΔT_{adi} comes from the response of *s*-CE induced by GNS. As illustrated in the main text, the ΔT_{adi} of 1GNS/HH observed via the direct temperature measurement was 20% higher than that of HH, and this value is consistent with that of 22% derived from the statistic results of the mean square end-to-end distance. The comparison

between the theoretical value and the measured value highlights the importance of enhancing the overall CE response of the system through the nanofiller-induced collaboration between the *s*-CE and the original *e*-CE.

Supplementary Discussion 2. COP_{mat} of 1GNS/HH during *e*-CE cycles.

At zero temperature span, the COP_{mat} of 1GNS/HH near room-temperature was determined by calculating the ratio of the cooling energy per unit mass (Q/m) to the input work on the unit mass sample (W/m):

$$COP_{mat} = \frac{\frac{Q}{m}}{\frac{W}{m}} = \frac{\int_{T_c}^{T_a} c_p dT}{\frac{1}{\rho l_0}\oint \sigma dl} \qquad \text{Supplementary Equation (14)}$$

where, ρ is the sample density, l_0 is the initial sample length, l is the sample length. $\oint \sigma dl$ was calculated from the hysteresis of stress-displacement characteristics as displayed in Supplementary Fig. 11 by integrating stress (σ) over displacement (l) for stretching and recovery cycles. $Q/m = \int_{T_c}^{T_a} c_p dT$ can be calculated by integrating area of specific heat capacity curves from T_c to T_a in Fig. 1d. The extensive internal stress generated in the modeling process of GNS/HHs required additional work to eliminate this irreversible effect, resulting the extremely high values of the hysteresis of stress-displacement and W/m and the low values of COP_{mat} during the first 10 cycles. The hysteresis of stress-displacement curves, $|\Delta T_{adi}|$ on the cooling process, W/m, Q/m as well as the COP_{mat} gradually stabilized after the first 10 cycles (Supplementary Fig. 11-12). It can be considered that the internal stress was basically excluded via the stretching recovery process.

During the stable cycles (Fig. 1b), the temperature changes between P1-P2 (ΔT_H) is higher than that of P3-P4 ($|\Delta T_{adi}|$). This is mainly due to the fact that the polymer in this work only undergoes

conformational changes (without first-order transition) during deformation. Therefore, the differences between ΔT_H and $|\Delta T_{adi}|$ during the heating and cooling processes are mainly caused by the internal friction within the molecular chains. The irreversible heat generated by internal friction could increase the temperature change caused by conformational arrangement during the heating process and decrease the conformation-induced temperature change during the cooling process. The irreversible heat generated by internal friction could be considered as the thermal hysteresis (ΔT_{hys}) of this material, and calculated as $\Delta T_{hys}=\frac{1}{2}(\Delta T_H - |\Delta T_{adi}|) = 3.6$ K. The ΔT_{hys} represents the irreversible portion of material during the *e*-CE cycle. Therefore, reducing ΔT_{hys} helps improve system efficiency, which can be achieved by adding plasticizers or other methods to reduce internal friction.

Supplementary Discussion 3. *s*-CE in different nanofillers-polymers systems.

Nanofillers such as multi-walled carbon nanotubes (MCNT) and conductive carbon black (CB) could also contribute to forming the π-π stacking owing to the sp^2 conjugated network. Therefore, the *s*-CE was also found in MCNT/HHs and CB/HHs (Supplementary Fig. 15a and Fig.1a). Among them, GNS/HHs and MCNT/HHs showed a high *s*-CE at a lower loading, which may be due to the higher aspect ratio of MCNT and GNS compared to CB. Nanofillers with higher aspect ratios could connect more *s*-CE units, but it could also lead to a more easily formed nanofiller aggregate network. Hence, the onset of the shear-damping transition would occur at a reduced nanofiller loading. For different matrices (Supplementary Fig. 15b and Fig. 1a), the *e*-CE of HH and LH, with different chain-length uniformity and the same higher orientation mobility, increased by 20% at most. While that of HL, with lower molecular chain orientation mobility, increased by 30%. This illustrates that the shear action has a better promotion effect on the network with weak mobility. In fact, in a system with low chain-length uniformity, the elongated short chain will limit

the further elongation of the long molecular chains. This restriction still exists under the shear drive of the nanofillers.

Supplementary Discussion 4. The double-unit cooling device using 1GNS/HH refrigerant.

Supplementary Fig. 16a illustrates the physical diagrams of the double-unit cooling system. The driving wheels of the two cooling units (U1 and U2) penetrated the cooling units from the backsides and were connected to the right-angle conversion gearbox through two couplings. The two driving wheels of two units could generate coaxial rotation under the drive of the motor. As shown in Supplementary Fig. 16b, two ends of the sample were fixed at the bottom and the driving wheel of the cooling unit respectively. As shown in Supplementary Fig. 16c, the sample was prepared by heat sealing of 1GNS/HH sheet (S1) and ultra-high molecular weight polyethylene fibers (S2). Since the modulus of S2 is two orders of magnitude higher than that of S1, it can be considered that S2 is a traction material for S1 without deformation or eCE effect during cycles. In the cooling unit, the effective length of S2 was 6 times that of S1. The refrigerant thereby was driven by the driving wheel to realize the elongation and recovery. In this work, the heat sink was a radiator which maintained around room-temperature and could dissipate the hot thermal energy carried by the hot outlet water until room-temperature. As shown in Supplementary Fig. 16d, the heat source was an electric heater (PTC100, rated voltage of 24V, the electrical resistance of 100 Ω at 20 ^{o}C) which was maintained in an insulated reservoir. The outlet water (m_r) at the cold end of the cooling units was pumped and stored in the insulated reservoir (made of PVDF), thus cooling the electric heater. Typical parameters of a single refrigerant sample and the corresponding heat transfer fluid are listed in Supplementary Table 2-3.

In addition, the periodic changes of the servo motor are controlled by a programmable driver. The flow rate and operating frequency of the one-way pumps are controlled by a programmable microcontroller. K-type thermocouples are used to monitor the temperature of the heat transfer fluid in the heat sink and the insulated reservoir. A digital power meter (PM9808, Sampling rate 8000 Hz, Dongguan Napui Electronic Technology Co., Ltd.) was used to evaluate the input power value (P_{in}) of the entire system as a function of time (t).

Due to the flow rate (V_{dot}) of the heat transfer water could impact the heat transfer duration (t_{cycle}) and the temperature change ($\Delta T = T_{out} - T_{amb}$), it is necessary to determine the optimal V_{dot} first. At the strain level from 100% to 500%, Supplementary Fig. 17a illustrates the ΔT of the outlet water at the cold end (heat source in Fig. 4a is ambient) as a function of t during a single cycle at different flow rates. The outlet water temperature at the cold end first decreases to a peak-to-valley value and then returns to the initial temperature during the heat exchange process. The peak-to-valley values of the cold end water decrease first and then increase with the increasing of V_{dot}. And t_{cycle} continuously decreases. When V_{dot} is 1.2 mL min^{-1}, the peak-to-valley value reaches −1.4 K. The following formula can be used to calculate the cold thermal energy (Q_{out}) exported from the cooling unit (U) during a single cooling cycle:

$$Q_{out} = \rho_{out} V_{dot} c_{p,out} \int \Delta T \mathrm{d}t \qquad \text{Supplementary Equation (15)}$$

where, ρ_{out}, $c_{p,out}$ is the density and specific heat capacity of the heat transfer water. $\int \Delta T \mathrm{d}t$ can be obtained from the integral area of the ΔT *versus* t curves in Supplementary Fig. 17a. As illustrated in Supplementary Fig. 17b, Q_{out} during a single cooling cycle reaches a maximum value when the V_{dot} is 1.2 mL min^{-1}. The difference values of Q_{out} are mainly caused by the friction loss and untimely heat exchange between the heat transfer water and the refrigerant. In this process, the specific cooling power (SCP) of the cooling unit (U) is defined by:

$$\mathrm{SCP} = \frac{Q_{\mathrm{out}}}{m t_{\mathrm{cycle}}} \qquad \text{Supplementary Equation (16)}$$

where, *m* is the mass of refrigerant.

When the thermal energy generated by a single operation of refrigerant is fully utilized, t_{cycle} is equal to the timescale for complete heat transfer (Supplementary Fig. 17a), so the frequency and t_{cycle} can be determined synchronously with the flow rate. In this case, all the caloric effect from the refrigerant can be utilized, and the system experienced the highest COP. Supplementary Fig. 17b shows the SCP of the cooling unit under a strain level from 100% to 500%. SCP reaches the maximum value of about 5.47 W g^{-1} when the V_{dot} is 1.2 mL min^{-1}. In addition, Supplementary Fig. 17c shows that the double-unit cooling device achieves the highest cooling power and temperature span at a flow rate of 1.2 mL min^{-1}, which is mainly due to a balance between the amount and the duration of the heat exchange process. At this V_{dot}, the heat transfer duration t_{cycle} is 6 s and the operating frequency is 0.167 Hz. Interestingly, increasing the cycling frequency when the thermal energy generated by a single operation of refrigerant is not fully utilized, the optimum t_{cycle} in terms of SCP can be less than the timescale for complete heat transfer. The theoretical Q_{out} and SCP values under different t_{cycle} and a constant flow rate of 1.2 mL min^{-1} can be calculated by integrating the water temperature change over different heat transfer timescales (Supplementary Fig. 17a). The theoretical COP under different t_{cycle} and a constant flow rate of 1.2 mL min-1 can be projected using the input work per cycle and the aforementioned integrated Q_{out}. As shown in Supplementary Fig. 17d, the calculated SCP reaches the maximum value of 9.7 W g^{-1} when the t_{cycle} is reduced to 2 s at a constant V_{dot} of 1.2 mL min^{-1}. However, due to the incomplete heat exchange of the refrigerant, the motor consumption increases by about threefold, leading to a lower theoretical COP_{sys}. Therefore, considering COP_{sys} as a priority, in this study we choose 6 s as the

t_{cycle}. A more comprehensive evaluation of the compromise between SCP and COP_{sys} should be conducted in the future.

A single-unit cooling device can be obtained by disconnecting the coupling between one cooling unit (U1 or U2) and the right-angle conversion gearbox. When the heat sink in Fig. 4a is maintained at room temperature and the heat source in Fig. 4a is maintained in an insulated reservoir, the coefficient of performance (COP_{sys}) for the overall single-unit cooling device and the double-unit cooling device on cooling are evaluated by:

$$\mathrm{COP_{sys}} = \frac{\dot{Q}_\mathrm{c}}{\dot{W}_\mathrm{in}} \qquad \text{Supplementary Equation (17)}$$

where, $\dot{Q}_c$ is the cooling power of the system which is equal to the power of the electric heater at stable T_{span}, $\dot{W}_\mathrm{in} = \frac{\int P_\mathrm{in}\mathrm{d}t}{t}$ is the average input power of the system during the recovery process which can be calculated by Supplementary Fig.18.

The work recovery efficiency (η) of the double-unit system compared to the single-unit cooling device, with the same mass of refrigerant, can be estimated by the ratio of the real recovery energy (W_r) to the ideal maximum recoverable work (W_i).[34] Considering a complete stretch-recovery cycle of a refrigerant sample, we obtain the following expression for η:

$$\eta = \frac{W_\mathrm{r}}{W_\mathrm{i}} \times 100\% = \frac{(2W_\mathrm{r,s} - 2W_\mathrm{r,m}) - (W_\mathrm{r,d} - W_\mathrm{r,m})}{W_\mathrm{i}} \times 100\%$$

$$= \frac{2W_\mathrm{r,s} - W_\mathrm{r,d} - W_\mathrm{r,m}}{W_\mathrm{i}} \times 100\% \qquad \text{Supplementary Equation (18)}$$

where, $W_{r,s}$ and $W_{r,d}$ are the real consumed energy for single-unit and double-unit devices during a t_{cycle} respectively. $W_{r,m}$ is the energy loss caused by the motor within a rotation process. W_i is the ideal maximum recoverable work for a complete stretch-recovery cycle of a refrigerant sample.

$W_{r,s}$ = 4.02 J and $W_{r,d}$ = 4.14 J can be obtained by multiplying the average power (Supplementary Fig. 18a, 18b) of the single-unit and double-unit device by t_{cycle}, respectively. $W_{r,m}$ = 2.39 J can be obtained by multiplying the average power of the no-load motor by t_{cycle} (Supplementary Fig. 18c). $W_{r,m}$ is considered constant for both the single-unit and double-unit devices. In the double-unit system, two refrigerant samples are engaged in the operational process during a rotation of the motor throughout the t_{cycle}. Throughout this period, the two refrigerant samples undergo elongation and contraction, respectively. Consequently, $W_{r,d}$ can be conceptualized as the sum of the work consumed by a refrigerant sample through a complete stretch-recovery cycle and the energy loss (equal to $W_{r,m}$) of the motor during a single rotation. In the single-unit system, a refrigerant sample is engaged in the operational process during twice rotations of the motor throughout the $2t_{cycle}$. Throughout this period, a refrigerant sample undergoes one complete stretch-recovery cycle. Consequently, $2W_{r,s}$ can be conceptualized as the sum of the work consumed by a refrigerant sample through a complete stretch-recovery cycle and the energy loss (equal to $2W_{r,m}$) of the motor during twice rotations. Comparing these two mechanisms, W_r = 1.51 J (the numerator of Supplementary Equation (18)) is the energy consumption difference of the refrigerant samples under these two kinds of systems, where both mechanisms drive a refrigerant sample to undergo a complete stretch-recovery cycle. This consumption difference could be considered as the real recovered energy generated by the double-unit operating mechanism. On the other hand, W_i could be considered from the material level. As illustrated in Supplementary Fig. 20, when assuming that the unloading work of the refrigerant could be completely reused, the integrated area of the mechanical hysteresis (red-shaded area) is the energy consumed per unit volume of the refrigerant for a complete stretch-recovery cycle. This consumption represents the loss of work at the material level, which is caused by the material properties (internal friction). In this case, the integral area of the recovery curve (blue-shaded area) can be considered as the maximum energy that can be

recovered by the system during a stretch-recovery cycle for a unit volume of refrigerant. At the strain range from 100% to 500%, the ideal maximum recoverable work (denominator in Supplementary Equation (18), W_i) can be obtained as W_i = 2.68 J by multiplying the integral area of the blue shaded in Supplementary Fig. 20. According to Supplementary Equation (18), η = 56.3% of the double-unit cooling system under stable cycling can be obtained.

The principle of the work recovery function of the double-unit device can be analyzed through the force distribution of refrigerants. Considering complete stretch-recovery cycles for two refrigerant samples in both cooling units, Supplementary Fig. 19a illustrated the changes of the force applied to the refrigerants during the operation of the double-unit device. During P1 to P2, the refrigerant in the U1 unit of the double-unit device is stretched and the refrigerant in the coaxial connected U2 unit synchronously shrinks. During P3 to P4, the refrigerant in the U1 unit of the double-unit device gradually shrinks and the refrigerant in the coaxial connected U2 unit is synchronously stretched. Thus, the force required to drive the refrigerants in both units during a single stable cooling cycle, can be considered as the difference value between the recovery forces and the external tensile forces of refrigerants in U1 and U2 (Supplementary Fig. 19b). At the strain range from 100% to 500%, the work (material level) required to drive the refrigerants in the double-unit device during a single stable cooling cycle, can be obtained as about 2.5 J by integrating the blue shaded area in Supplementary Fig. 19b. And the work (material level), when driving same mass of refrigerant in single-unit devices (without work recovery), can be obtained as about 6.8 J by integrating the force-displacement curve of the stable stretching process in Supplementary Fig. 19a.

Supplementary Discussion 5. Comparison of the application-oriented cooling performances and the cooling devices among polymer and SMA.

The *e*-CE activities and the large-deformation feature of polymers, which are discussed in the main text, are the most fundamental challenges for polymers to move toward solid-state cooling applications. In addition to the above performances, other cooling performances of different materials and associated device performances are summarized in Supplementary Table 4, which would help to classify the current work.

Commercial air conditioners, based on vapor-compression cooling technology, generally need to be recharged with additional refrigerant approximately every 5 years. This is mainly caused by the gradual leakage of gas refrigerant. For solid-state cooling applications, the frequency of the replacement of solid refrigerant is determined by the fatigue life of refrigerant materials and the operation frequency of the cooling device. With 0.167 Hz operation for a usage modality of 12 hours per day and 180 days per year,[31] 6.5×10^6 cycles correspond to 5 years, which could meet the service life of commercial applications. NiTi alloy almost meets this requirement as shown in Supplementary Table 4.[30,31] For the large-deformation refrigerants, only the cycling fatigue life of NR for *e*-CE cooling has been systematically studied.[32] Without considering the weather resistance, the result shows a high fatigue life of up to 1.7×10^5 cycles for NR samples with little degradation of *e*-CE properties, constituting an important demonstration of cooling application. The fatigue life of large-deformation refrigerant has not been determined further, which is due to the focus of current research being on the design and testing of the cooling device.[19,20,26] For 1GNS/HH in this work, $\sim10^3$ cycles of fatigue life are achieved from testing of the cooling device. At the materials level, Supplementary Fig. 21 shows little degradations of the mechanical strength and *e*-CE activity of 1GNS/HH after the first 10 cycles. Moreover, the fatigue life of SEBS can be improved

to 8.8×10^4 cycles through further modifications, which makes it possible to optimize the material composition to meet practical requirements.[33] The mechanism of fatigue fracture of SEBS and the corresponding performance improvements still require further research.

Another challenge for polymers is heat transfer. As illustrated in Supplementary Table 5, GNS/HHs possess the maximum thermal conductivity of 0.4853 W m^{-1} K^{-1}, which is two orders of magnitude smaller than that of alloy materials. This means that the heat transfer rate of polymer is lower than that of alloy under the conditions of the same shape of refrigerant and the same heat transfer medium. As illustrated in Supplementary Table 4, when the heat transfer medium is water and the shape of the refrigerant is bulk tube,[26, 30] the low thermal conductivity further hinders the operation frequency and specific cooling power of the associated polymer-based cooling device in order to ensure adequate heat exchange. In this case, the polymer-based cooling device could still maintain a higher temperature span. In this work, water flow is used as a heat transfer medium to extract the cooling energy generated from bulk refrigerant. As another major advantage of this configuration being the scalability of the refrigerant, it is possible to increase the mass of refrigerant, thereby increasing the cooling power without significantly altering the device structure. Another configuration of the cooling device uses a thinner refrigerant to transfer heat through solid-solid contact.[20,28] This configuration can significantly reduce the time required for heat transfer because of its high specific surface area, thereby achieving higher operating frequency and specific cooling power. This makes the operation frequency of polymer-based cooling devices comparable to that of alloy-based devices while achieving a specific cooling power of up to 20.9 W g^{-1}. However, the cooling power of the polymer-based cooling device is much lower under this configuration. This is because specific cooling power is related to both operation frequency and sample shape (specific surface area). As the value of specific cooling power does not take into

consideration of the mass or volume factors associated with the change of specific surface area, any changes in heat transfer caused by the mass/volume factors or scalability have not been considered. In summary, to improve the overall performance of polymer-based cooling devices, it is necessary to further combine the reduction of the heat transfer period with the increase of refrigerant mass.

Supplementary Figures

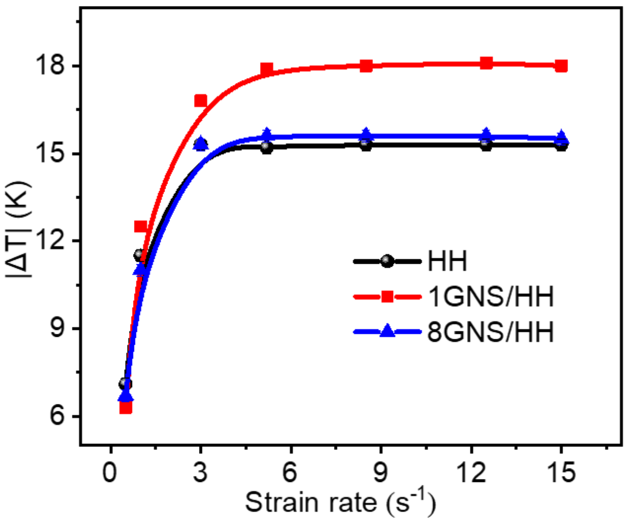

Supplementary Fig. 1. Surface |Δ*T*| of selected GNS/HHs as a function of strain rate. All samples were measured on the *e*-CE cooling process with a constant strain of 600% at room temperature.

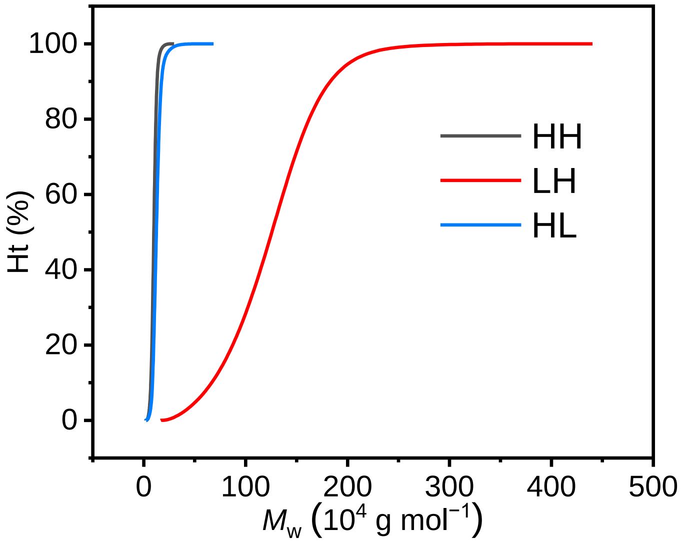

Supplementary Fig. 2. GPC curves of composite matrices. The maximum $M_{w\text{-max}}$ appears at Ht=90%. The minimum $M_{w\text{-min}}$ appears at Ht=10%. The weight average molecular weight of HH, LH, and HL are 100,921 g mol^{-1}, 1,271,743 g mol^{-1}, and 129,698 g mol^{-1}, respectively.

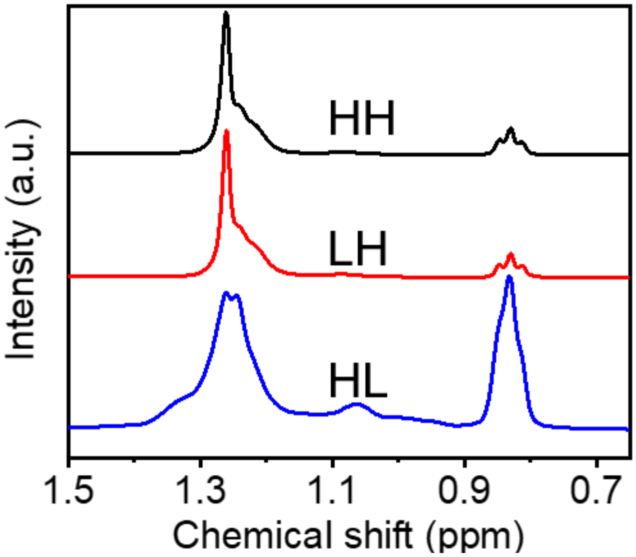

Supplementary Fig. 3. ^{1}H-NMR analysis of the matrices of composites.[5]

The wide signal peaks around 1.26 ppm were attributed to the hydrogen protons of CH_2 and CH in the polyethylene backbone and the ethyl side group, and the hydrogen proton resonance of methyl groups in the ethyl side group occurred at approximately 0.82 ppm. Et/C values of the SEBS matrix can be calculated from the relative areas of signal peaks at approximately 1.26 ppm and 0.82 ppm according to the following equation:

$$\text{Et mol\%} = \frac{\frac{1}{3} I_{0.82}}{\frac{1}{3} I_{0.82} + \frac{1}{2} (I_{1.26} - \frac{2}{3} I_{0.82} - \frac{1}{3} I_{0.82})} = \frac{2 I_{0.82}}{3 I_{1.26} - I_{0.82}}$$

where, $I_{1.26}$ represents the integral area of signal peaks around 1.26 ppm. $I_{0.82}$ represents the integral area of signal peaks around 0.82 ppm. $\frac{1}{3} I_{0.82}$ in the numerator represents the amount of ethyl side-group. $\frac{1}{3} I_{0.82}$ in the denominator represents the amount of the carbon atoms of CH groups in the PEB backbone. $\frac{1}{2} (I_{1.26} - \frac{2}{3} I_{0.82} - \frac{1}{3} I_{0.82})$ in the denominator represents the amount of the carbon atoms of CH_2 groups in the PEB backbone.

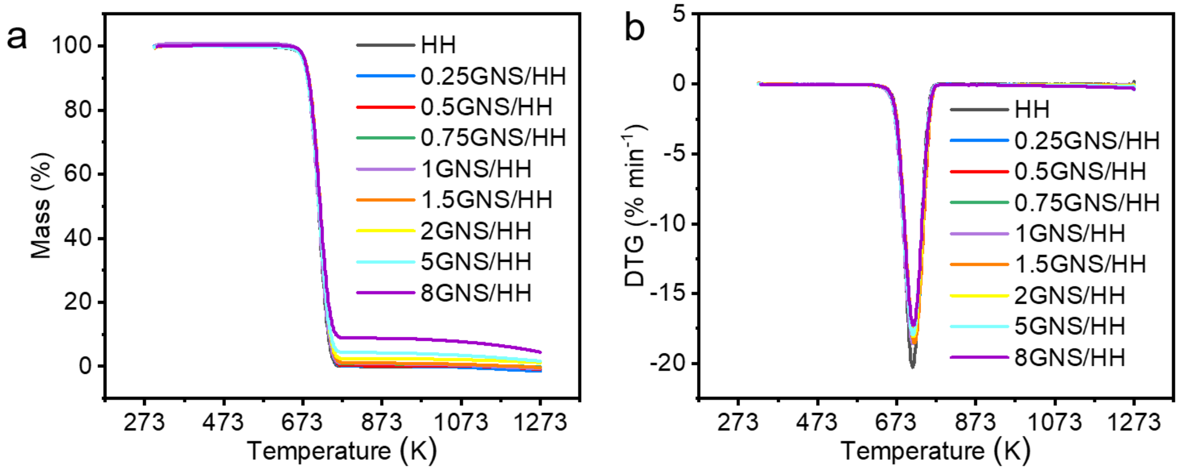

Supplementary Fig. 4. TGA and DTG curves of GNS/HHs. a TGA curves show that no mass loss before the onset decomposition temperature of 678 K. **b** DTG curves show that only one decomposition peak related to the matrix appears at 708 K.

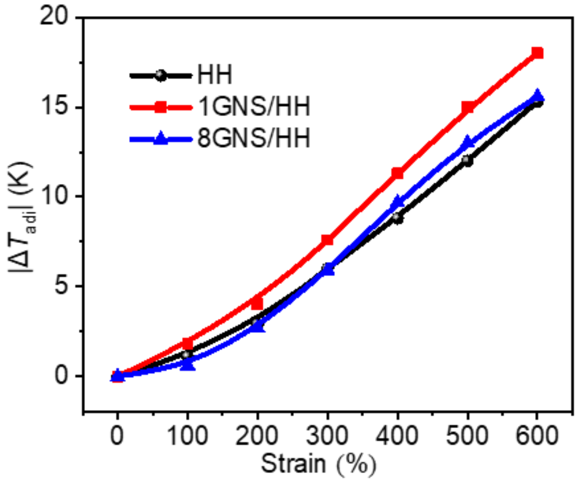

Supplementary Fig. 5. |ΔT_{adi}| of selected GNS/HHs as a function of strain during the cooling process. All data were collected at a strain rate of 15 s^{-1}.

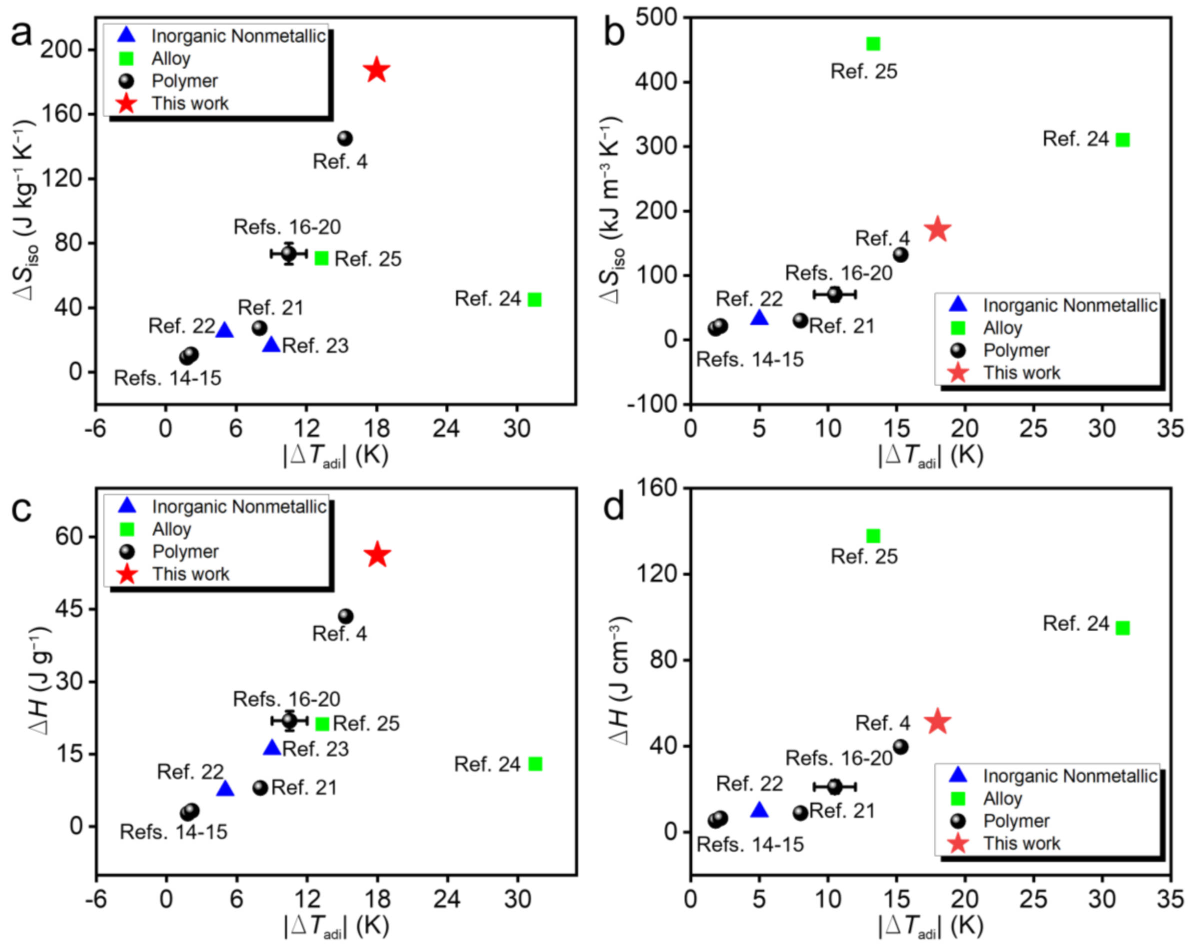

Supplementary Fig. 6. *e*-CE activity compression of 1GNS/HH with state-of-the-art elastocaloric materials near room temperature. a Massic quantity for isothermal entropy change. **b** Volumetric quantity for isothermal entropy change. **c** Massic quantity for enthalpy change. **d** Volumetric quantity for enthalpy change.

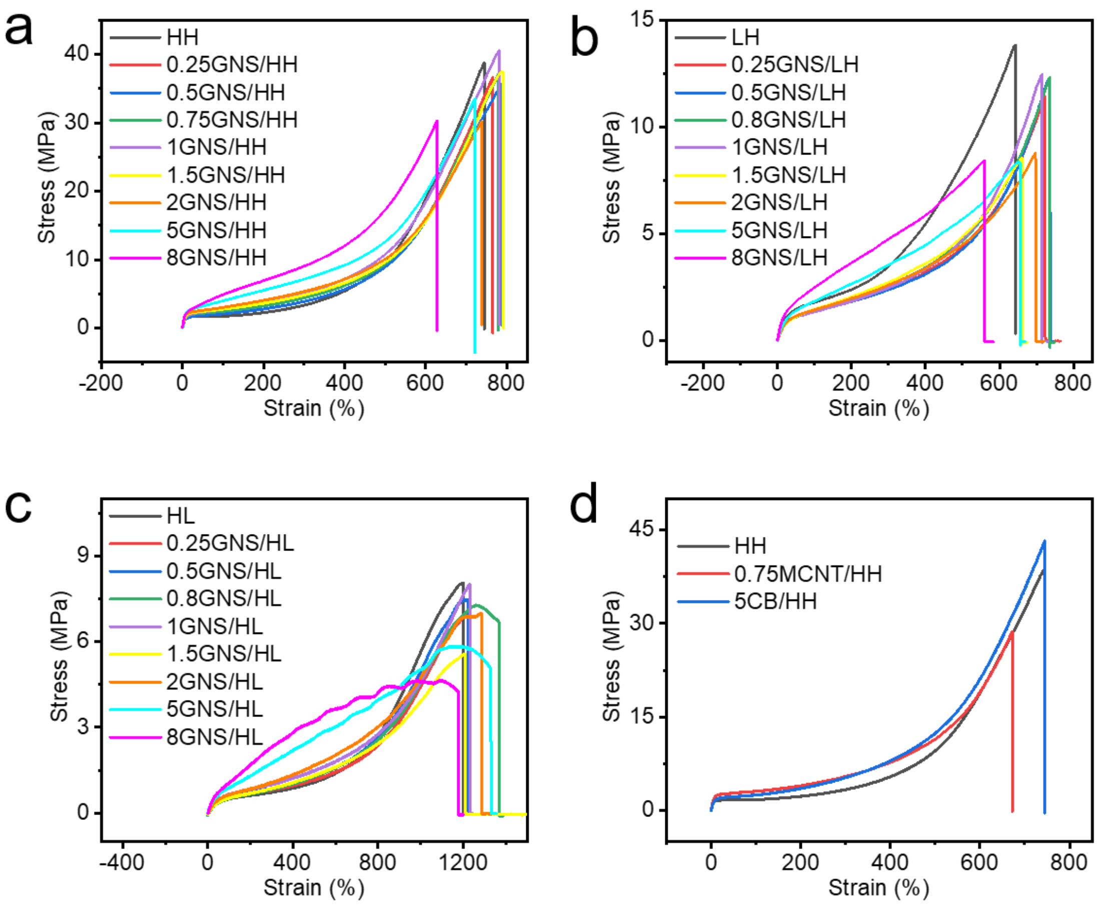

Supplementary Fig. 7. Stress-strain curves of the nanofillers/SEBS composites. a Stress-strain curves of composites with the same HH matrix and different content of GNS. The fracture strains of the samples are about 700%. **b** Stress-strain curves of composites with the same LH matrix and different content of GNS. The fracture strains of the samples are about 700%. **c** Stress-strain curves of composites with the same HL matrix and different content of GNS. The fracture strains of the samples are about 1200%. **d** Stress-strain curves of composites with the same HH matrix and different nanofillers. The fracture strains of the samples are about 700%.

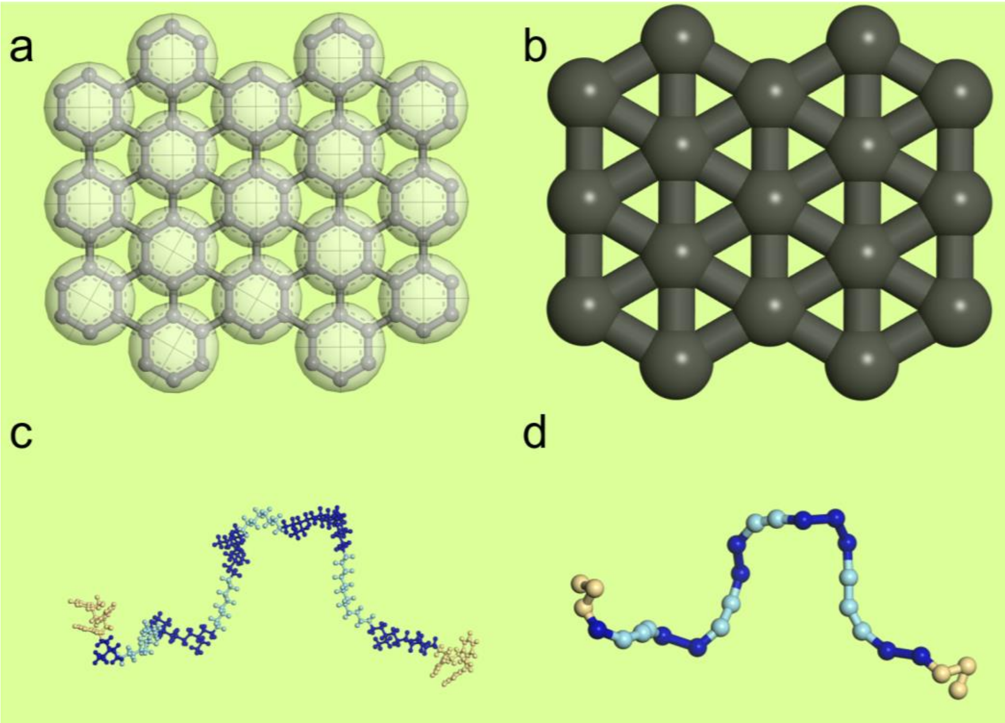

Supplementary Fig. 8. Structure and the coarse-grained (CG) model of the matrix molecular chain and GNS. a Structure of GNS. **b** CG of GNS. **c** Structure of matrix molecular chain. The yellow parts represent PS segments, the water-blue parts represent the ethylene segments and the navy-blue parts represent the butylene segments. **d** The CG model of the matrix molecular chain.

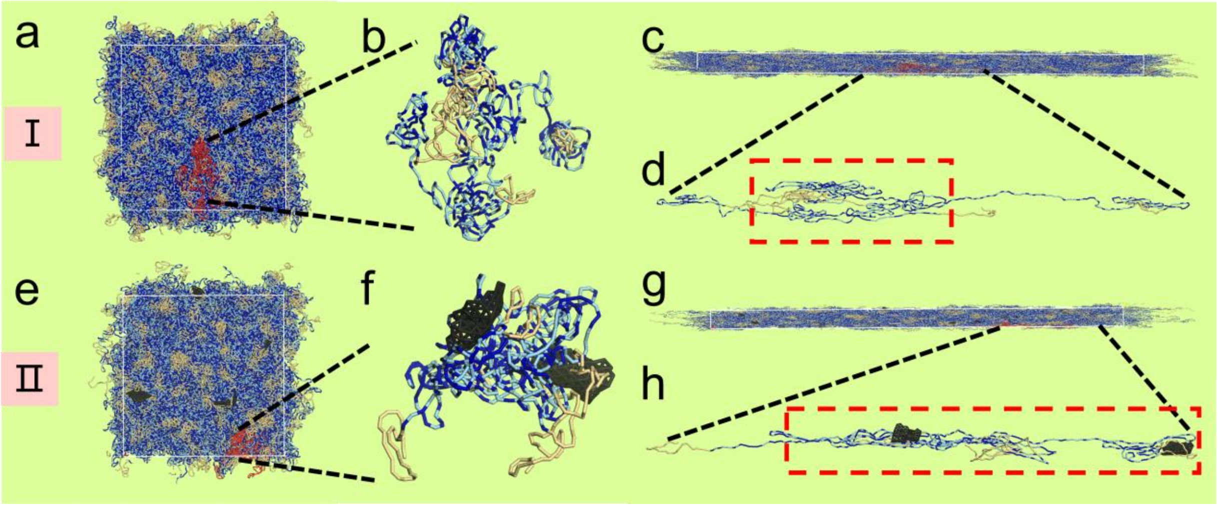

Supplementary Fig. 9. Minimum elastic unit in GNS/HHs during the stretching process. a CG model for HH without GNS at initial state. **b** A selected minimum elastic unit for HH at the initial state. **c** CG model for HH without GNS at the elongation state. **d** Same elastic unit in **Supplementary** Fig. 9b under deformation. **e** CG model for 1GNS/HH at initial state. **f** A selected minimum elastic unit for 1GNS/HH at initial state. **g** CG model for 1GNS/HH at elongation state. **h** Same elastic unit in **Supplementary** Fig. 9f under deformation.

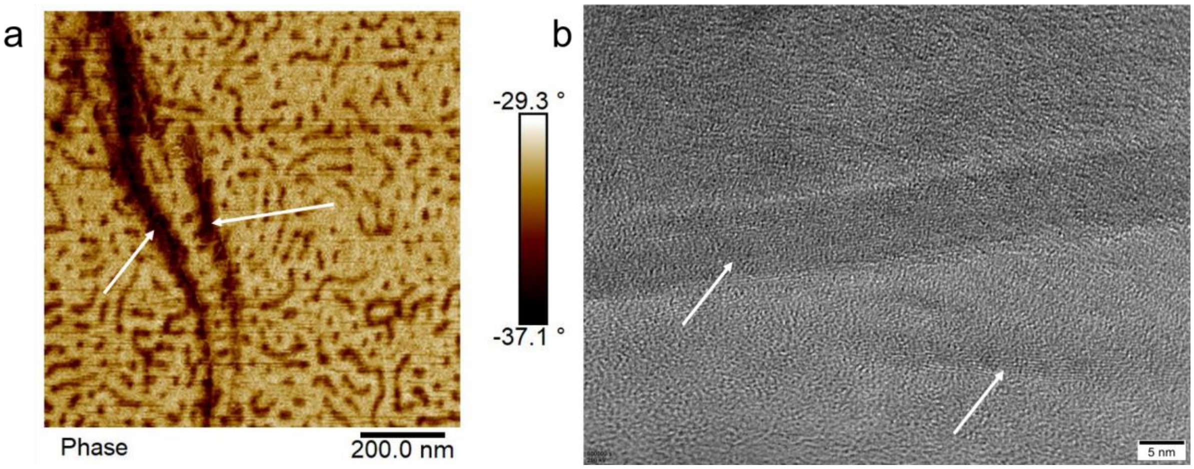

Supplementary Fig. 10. Lower magnification of TEM image and AFM phase diagram of 1GNS/HH. The white arrows point to the GNS nanofillers.

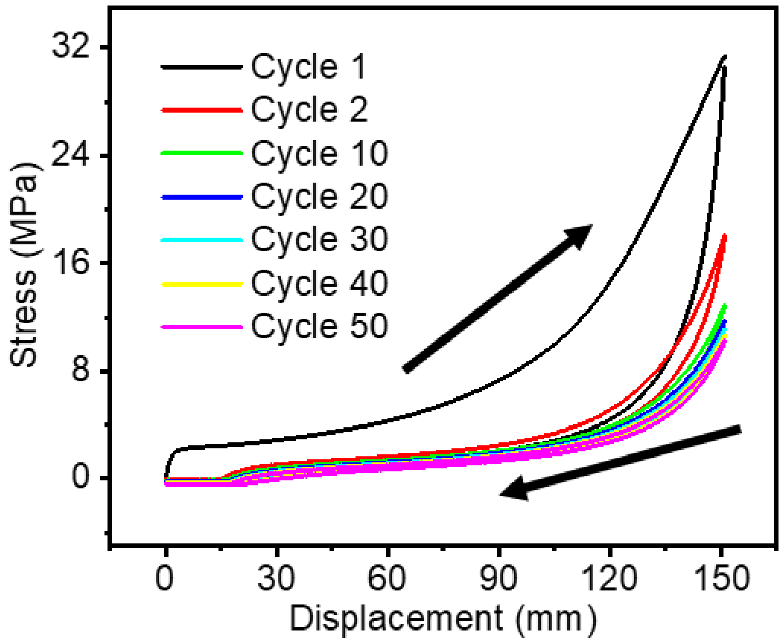

Supplementary Fig. 11. Stress-displacement characteristics of 1GNS/HH during 50 times stretching and recovery cycles. The black arrows point to the direction of strain development.

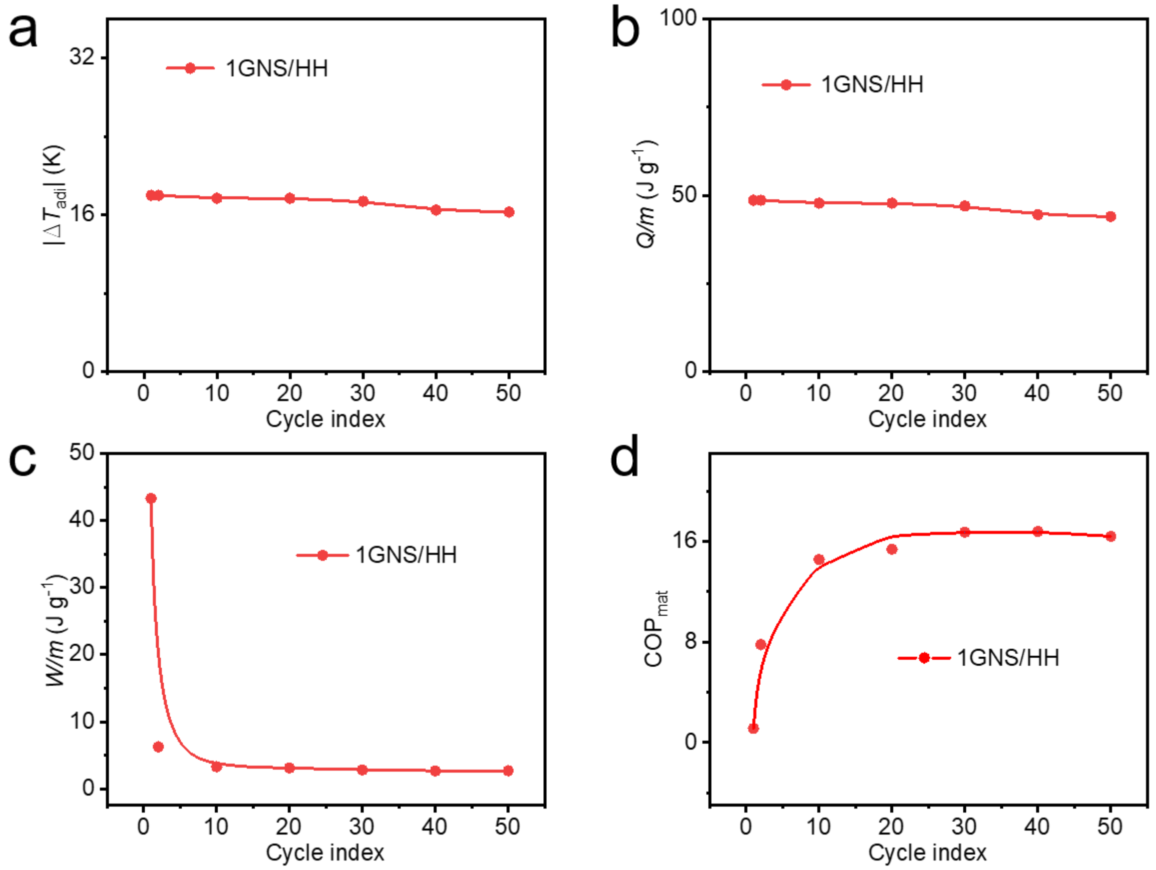

Supplementary Fig. 12. Cycling performance of 1GNS/HH near room-temperature. a Surface $|\Delta T_{adi}|$ evolution on cooling during *e*-CE cycles. **b** Cooling energy per unit mass during the *e*-CE cycles. c Input work per unit mass during *e*-CE cycles. **d** COP_{mat} of GNS/HHs during *e*-CE cycles.

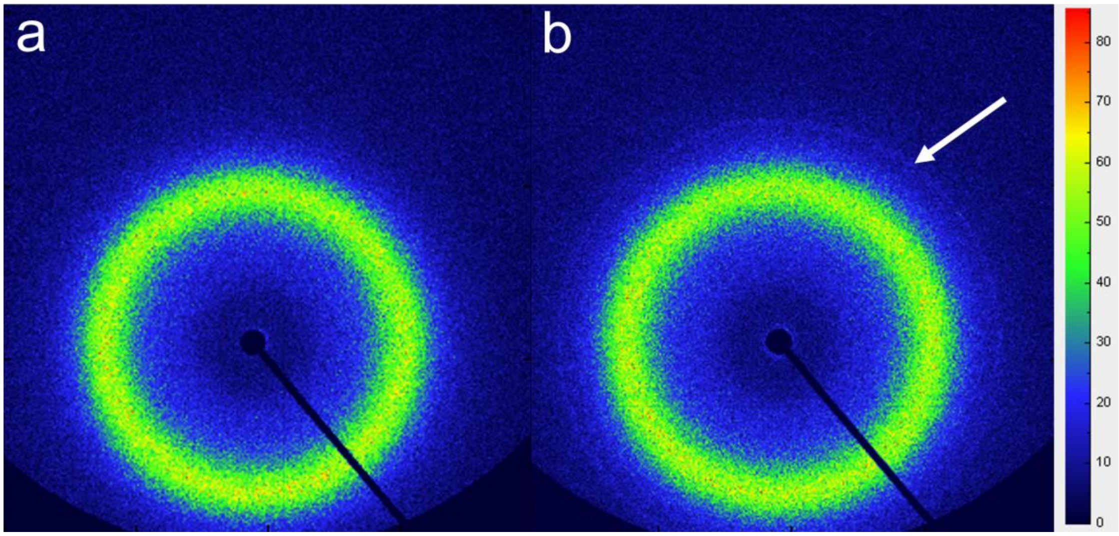

Supplementary Fig. 13. 2D WAXD patterns of GNS/HH when returned to the initial length. **a** HH. **b** 1GNS/HH. The white arrow in Supplementary Fig. 13b points to the (002) crystal plane of GNS.

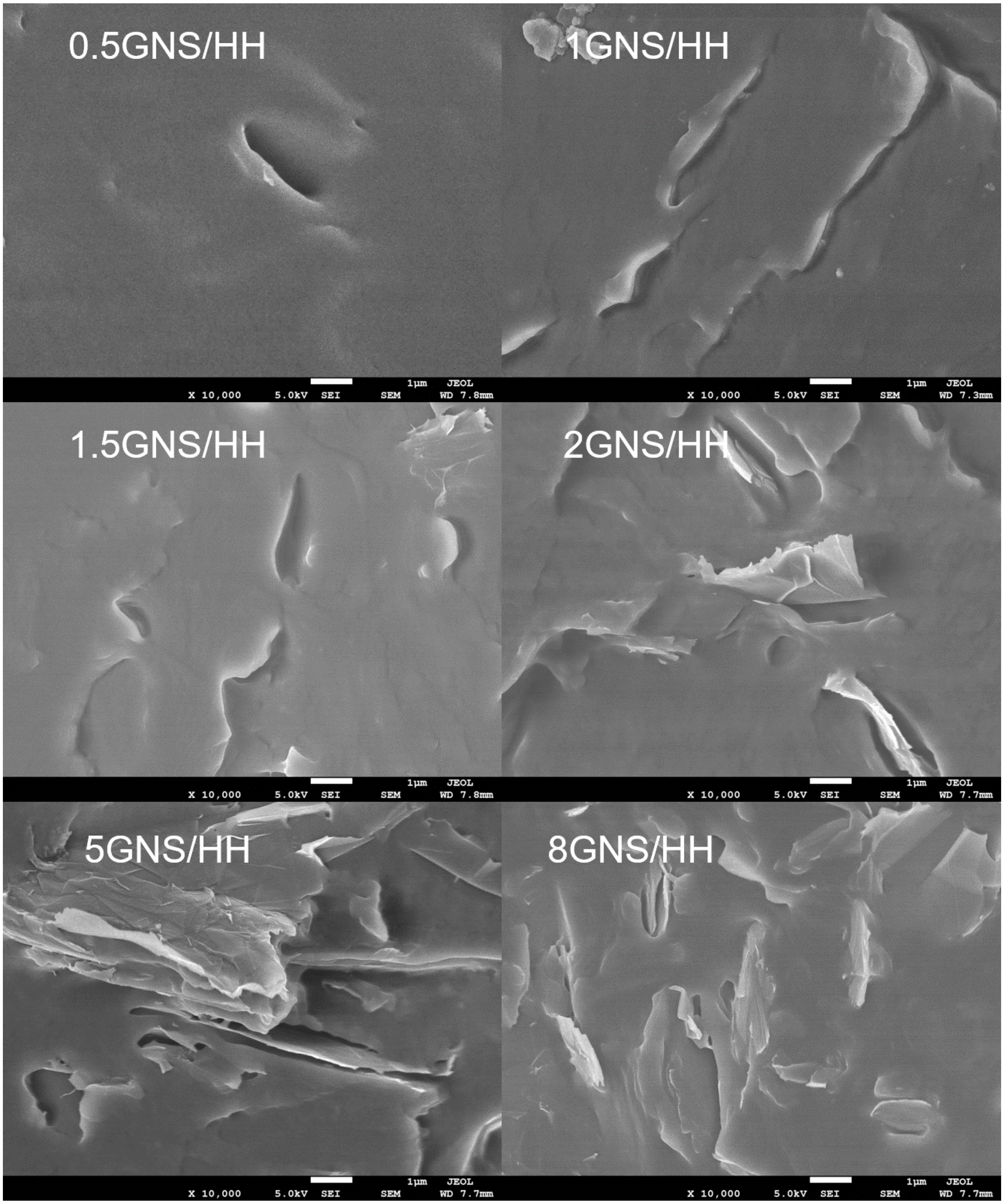

Supplementary Fig. 14. FE-SEM images for GNS/HHs. All composite samples were placed in liquid nitrogen overnight, and the cross section of those after brittle fracture was used to investigate the morphology.

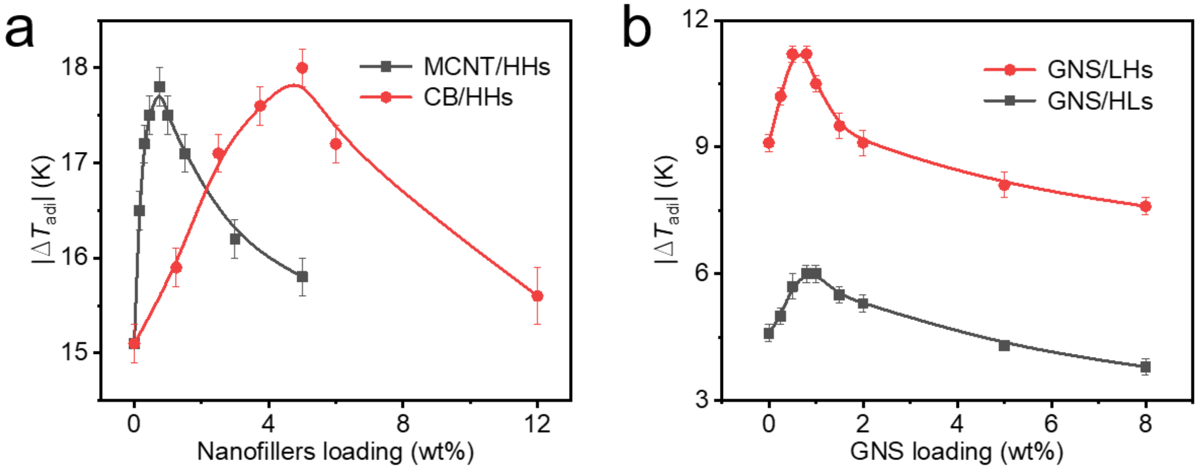

Supplementary Fig. 15. *s*-CE in different nanofillers-polymers systems. a $|\Delta T_{\mathrm{adi}}|$ *versus* the mass ratio of MCNT and CB in the same matrix HH during the cooling process. **b** $|\Delta T_{\mathrm{adi}}|$ *versus* the mass ratio of GNS in matrices of LH and HL on the cooling process. The error bars represent the average values of 6 tests under the same conditions.

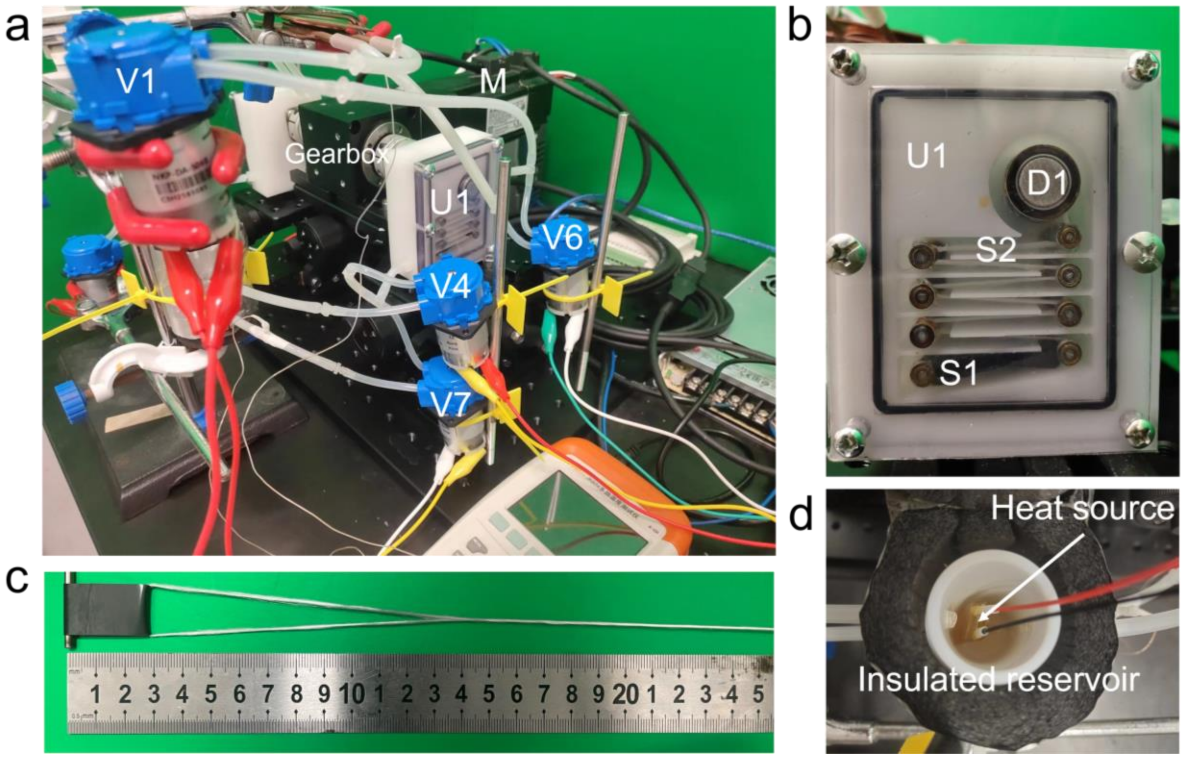

Supplementary Fig. 16. Physical diagrams of the double-unit cooling system, cooling device, and sample. a Double-unit cooling system. **b** Cooling unit with refrigerant equipped. **c** 1GNS/HH refrigerant connected with ultra-high molecular weight polyethylene fiber through heat sealing. **d** Electrical heater as a heat source is maintained in the PVDF insulated reservoir.

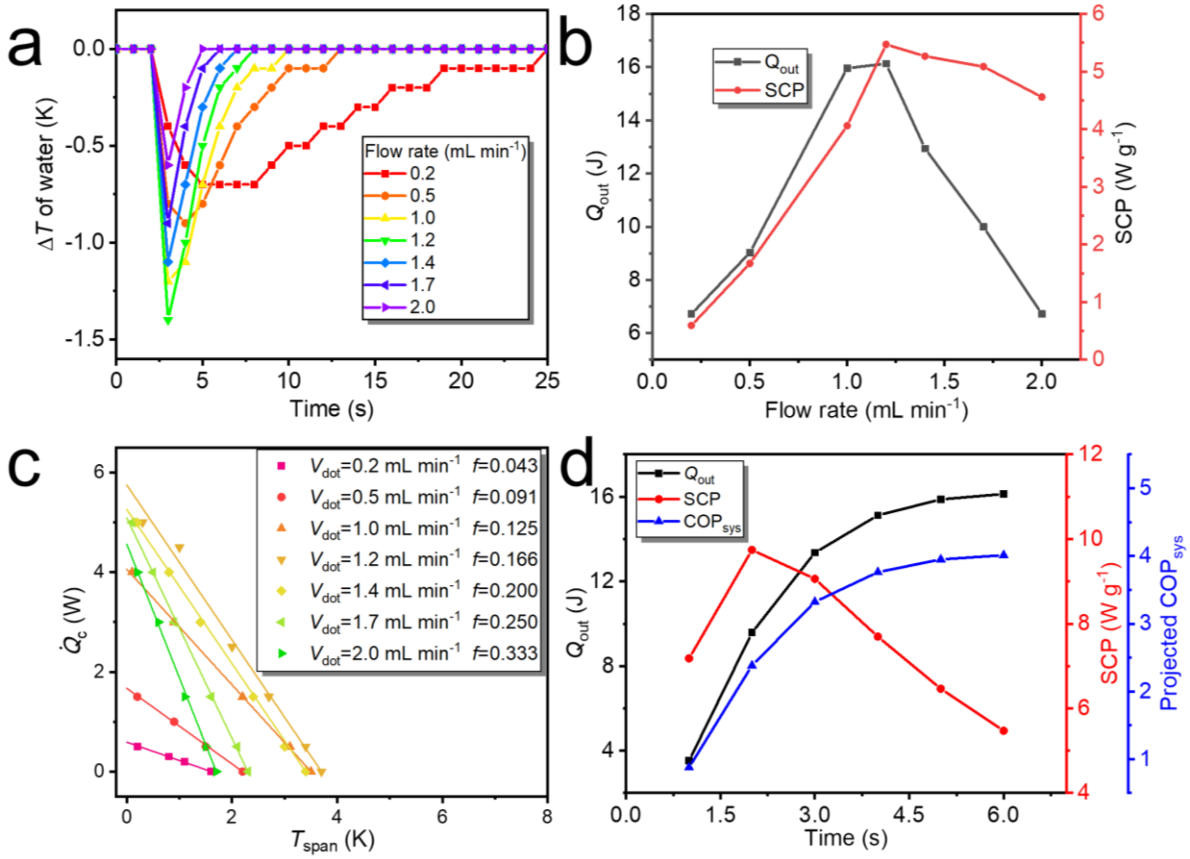

Supplementary Fig. 17. Basic performance characteristics of the cooling devices and refrigerants. a Δ*T* of water *versus* time under different flow rates when the heat source is ambient. **b** Q_{out} and SCP under different flow rates when the heat source is ambient. **c** Cooling power *versus* temperature span under different flow rates and operation frequencies. **d** Calculated Q_{out}, SCP, and projected COP_{sys} under different heat transfer timescales when the flow rate is constant at 1.2 mL min^{-1}.

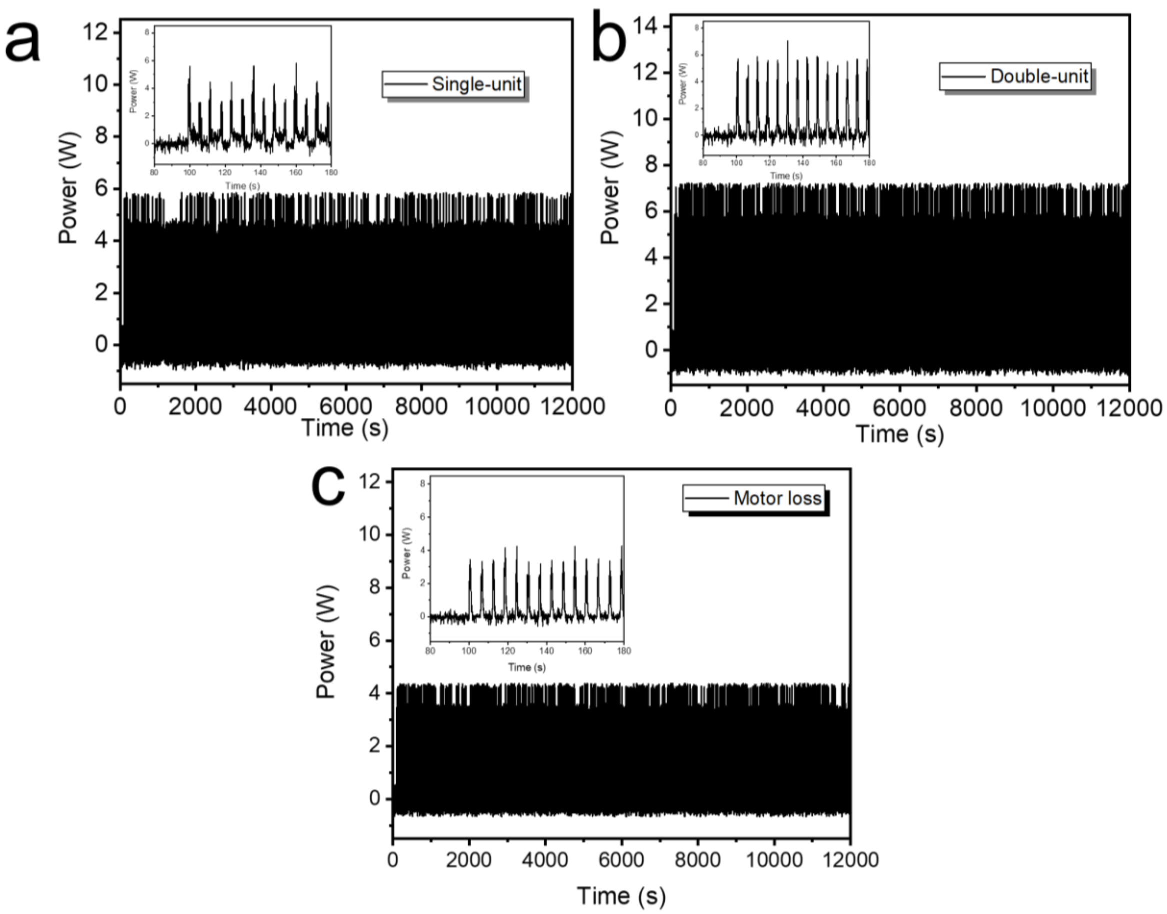

Supplementary Fig. 18. Power *versus* time of the cooling devices. a Power *versus* time of the single-unit devices (with one refrigerant sample) at a flow rate of 1.2 mL min^{-1}. **b** Power *versus* time of the double-unit devices (with two refrigerant samples) at a flow rate of 1.2 mL min^{-1}. **c** Power *versus* time without refrigerant sample load (disconnect the couplings of both U1 and U2).

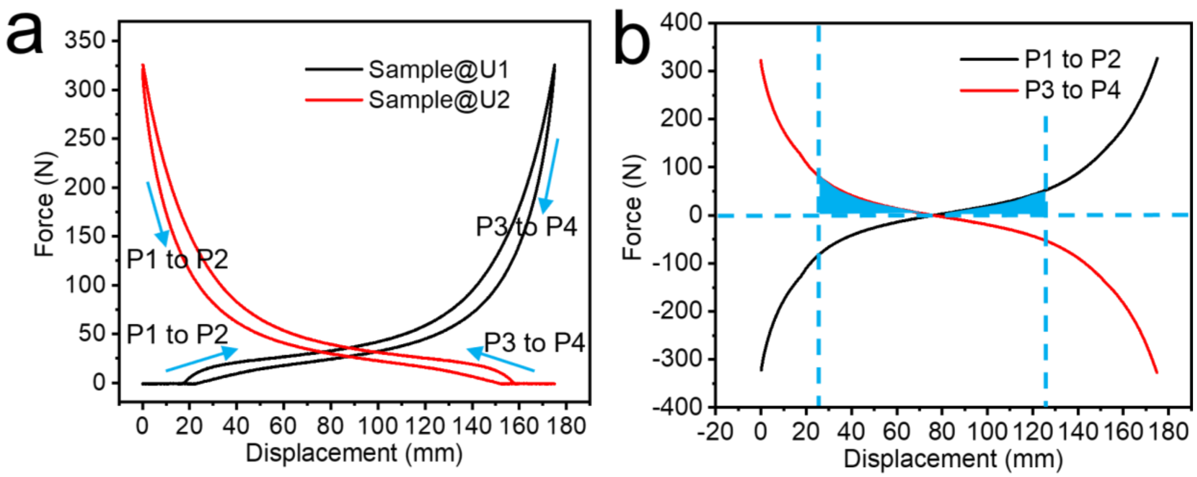

Supplementary Fig. 19. Force-displacement curves of refrigerants in the double-unit device under ideal conditions. a Changes of the force applied to the refrigerants during the operation of the double-unit deice. The blue arrows point to the force development during different cycle processes. **b** The force (material level) required to drive the refrigerants in the double-unit device. The horizontal axis represents the direction of motor operation.

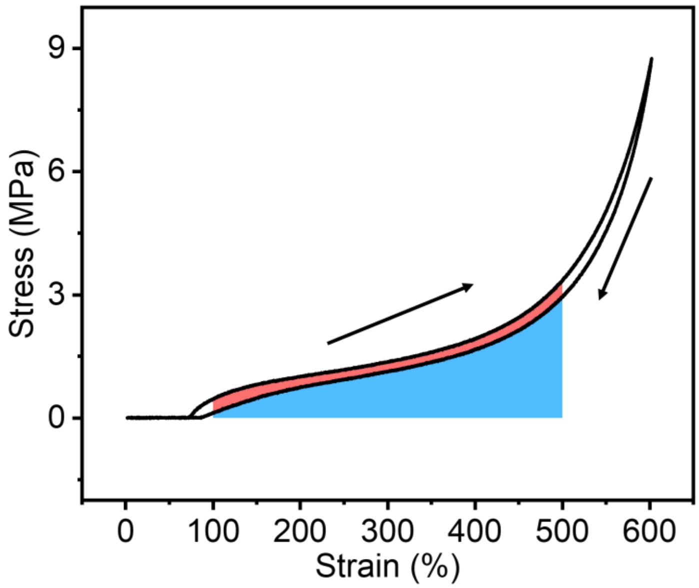

Supplementary Fig. 20. Stress-strain curves of 1GNS/HH during stable cycling. The black arrows point to the direction of strain development. The red shadow represents the integral area of the mechanical hysteresis. The blue shadow represents the integral area of the recovery curve.

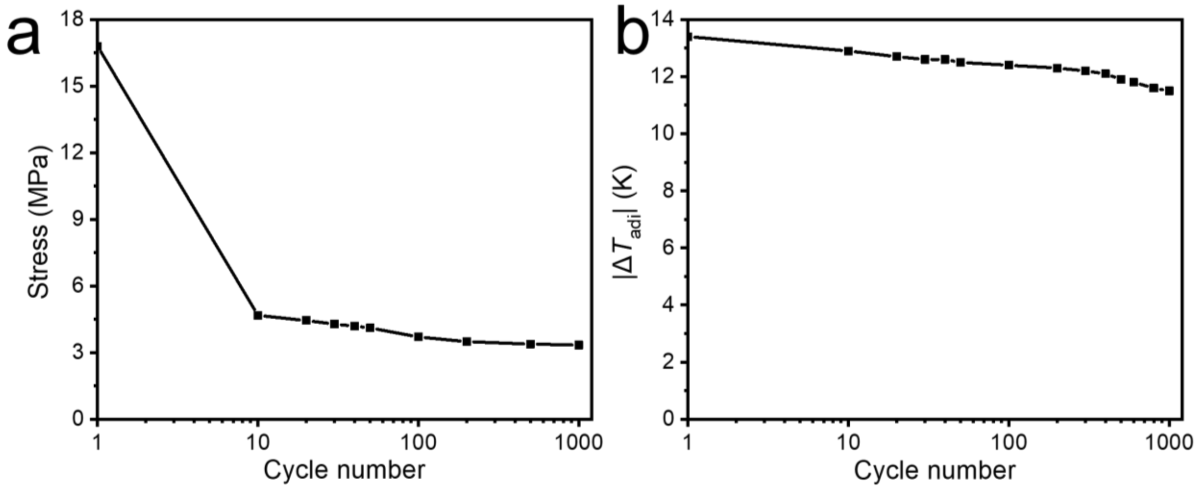

Supplementary Fig. 21. Fatigue performance of 1GNS/HH during a train level from 100% to 500%. **a** Mechanical strength. **b** ΔT_{adi} during the cooling process.

Supplementary Tables

Supplementary Table 1. Samples labeling, molecular properties of SEBS matrix, and types and mass fraction of nanofillers in the composites

Sample	MR [a] (10^4 g mol^{-1})	Et/C [b] (mol%)	Nanofillers	Mass fraction of nanofillers (wt%)
HH	7.9	11	GNS	0.00
0.25GNS/HH	7.9	11	GNS	0.25
0.5GNS/HH	7.9	11	GNS	0.50
0.75GNS/HH	7.9	11	GNS	0.75
1GNS/HH	7.9	11	GNS	1.00
1.5GNS/HH	7.9	11	GNS	1.50
2GNS/HH	7.9	11	GNS	2.00
5GNS/HH	7.9	11	GNS	5.00
8GNS/HH	7.9	11	GNS	8.00
0.15MCNT/HH	7.9	11	MCNT	0.15
0.3MCNT/HH	7.9	11	MCNT	0.30
0.45MCNT/HH	7.9	11	MCNT	0.45
0.75MCNT/HH	7.9	11	MCNT	0.75
1MCNT/HH	7.9	11	MCNT	1.00
1.5MCNT/HH	7.9	11	MCNT	1.50
3MCNT/HH	7.9	11	MCNT	3.00
5MCNT/HH	7.9	11	MCNT	5.00

Continue from the following Table.

Supplementary Table 1 (continued).

Sample	MR [a] (10^4 g mol^{-1})	Et/C [b] (mol%)	Nanofillers	Mass fraction of nanofillers (wt%)
1.25CB/HH	7.9	11	CB	1.25
2.5CB/HH	7.9	11	CB	2.5
3.75CB/HH	7.9	11	CB	3.75
5CB/HH	7.9	11	CB	5.00
6CB/HH	7.9	11	CB	6.00
12CB/HH	7.9	11	CB	12.00
LH	118.6	11	GNS	0.00
0.25GNS/LH	118.6	11	GNS	0.25
0.5GNS/LH	118.6	11	GNS	0.50
0.8GNS/LH	118.6	11	GNS	0.80
1GNS/LH	118.6	11	GNS	1.00
1.5GNS/LH	118.6	11	GNS	1.50
2GNS/LH	118.6	11	GNS	2.00
5GNS/LH	118.6	11	GNS	5.00
8GNS/LH	118.6	11	GNS	8.00
HL	8.8	33	GNS	0.00
0.25GNS/HL	8.8	33	GNS	0.25
0.5GNS/HL	8.8	33	GNS	0.50
0.8GNS/HL	8.8	33	GNS	0.80
1GNS/HL	8.8	33	GNS	1.00

Continue from the following Table.

Supplementary Table 1 (continued).

Sample	MR [a] (10^4 g mol^{-1})	Et/C [b] (mol%)	Nanofillers	Mass fraction of nanofillers (wt%)
1.5GNS/HL	8.8	33	GNS	1.50
2GNS/HL	8.8	33	GNS	2.00
5GNS/HL	8.8	33	GNS	5.00
8GNS/HL	8.8	33	GNS	8.00

[a] The molecular weight distribution range (MR) is defined as the difference value of the maximum molecular weight ($M_{w\text{-}max}$) and the minimum molecular weight ($M_{w\text{-}min}$) of a sample. For the convenience of quantification, the molecular weights with the integral distribution (Ht) of 0.1 and 0.9 on the integral distribution curve were regarded as $M_{w\text{-}min}$, and $M_{w\text{-}max}$ (Supplementary Fig. 2). The composite matrices with smaller MR values mean higher molecular chain-length homogeneity, and marked with the first "H".[4]

[b] The Et/C represented the molar ratio of the ethyl side group to the carbon atom in a polyethylene backbone of a soft PEB block. The composite matrices with smaller Et/C mean higher molecular chain orientation mobility and are marked with the second "H".[5] The molecular Et/C values in this work were verified by ^{1}H-NMR analysis (Supplementary Fig. 3).

When the composite matrix with high molecular chain-length homogeneity and high molecular chain mobility, the sample matrix is named "HH".

Supplementary Table 2. The parameters of the refrigerant and cooling device.

Symbol	Value	Explanation
l_s	30 mm	The length of the sample
w_s	18 mm	The width of the sample
t_s	1 mm	The thickness of the sample
m_r	50 g	The mass of the water in the insulated reservoir
V_{dot}	1.2 mL min^{-1}	The flow rate of the water
f	0.167 Hz	The operation frequency of motor motion

Supplementary Table 3. The parameters of 1GNS/HH near room temperature.

Sample	Specific heat ($J\ g^{-1}\ K^{-1}$)	Thermal conductivity ($W\ m^{-1}\ K^{-1}$)	Density ($g\ cm^{-3}$)	ΔT_{adi} (K)
1GNS/HH	~3	~0.2	~0.91	18.0

Supplementary Table 4. Performance comparison of *e*-CE cooling systems and materials.

Refrigerant						System					
Sample	Status	$\Delta\sigma$ (MPa)	ε (100%)	Cycles	Medium	COP_{sys}	T_{span} (K)	f (Hz)	$\dot{Q}_c$ (W)	SCP (W g^{-1})	Ref.
NiTi	Membrane Sheet	450	0.034	2×10^3	Water	3.5	15.3	0.25	4.64	0.8	27
TiNiFe	Membrane Foil	500	0.055	–	Cu metal	3.2	13	4	7.9	7.7	28
NiTi	Membrane Sheet	–	0.043	6×10^3	Water	–	19.9	–	–	–	29
NiTi	Bulk tube	1234	–	10^7	Water @	–	5.6	1.2	7.9	6.27	30
NiTi	Bulk tube	700	0.035	7.5×10^4	Water	6.85	22.5	0.071	260	0.3	31
NR	Bulk fiber	–	0-1 #	750	Water	–	0.7 ##	–	–	–	19
NR	Membrane balloon	*	**	10^3	Al metal	–	7.9 ***	1.1	0.75	20.9	20
NR	Bulk tube	1.5	3.5-5.5	3×10^4	Water	6	8.3	0.1	1.5	0.14	26
1GNS/HH	Bulk film	4	1-5	10^3	Water	8.3	3.7	0.167	5.0	5.47	This work

T_{span} is collected near zero $\dot{Q}_c$. $\dot{Q}_c$ and COP_{sys} are collected near zero T_{span}.

@ The heat is transferred by evaporation and condensation of water.

# Accompanying by isometrical twisting and untwisting at 15 turns/s. ## Assume that the temperature drop of the water is the T_{span} under zero $\dot{Q}_c$.

* 8.5 kPa of gas pressure. ** Balloon volume change from 115 to 200 cm^3. *** Temperature span between the hot and cold sides of a device.

Supplementary Table 5. Thermal conductivity of selected GNS/HHs at 298 K.

Sample	Thermal conductivity ($W\ m^{-1}\ K^{-1}$)
HH	0.1673±0.0001
1GNS/HH	0.1960±0.0002
8GNS/HH	0.4853±0.0003

Supplementary References

1. Gao, J. S., Liu, Z., Yan, Z. & He Y. A novel slurry bending method for a uniform dispersion of carbon nanotubes in natural rubber composites. *Results Phys.* **15,** 102720 (2019).
2. Enrique-Jimenez, P. et al. Control of the structure and properties of SEBS nanocomposites via chemical modification of graphene with polymer brushes. *Eur. Polym. J.* **97**, 1-13 (2017).
3. Han, X., Hu, J., Liu, H. & Hu, Y. SEBS aggregate patterning at a surface studied by atomic force microscopy. *Langmuir* **22,** 3428-3433 (2006).
4. Zhang, S. et al. Solid-state cooling by elastocaloric polymer with uniform chain-lengths. *Nat. Commun.* **13**, 9 (2022).
5. Zhang, S. et al. Polymer elastomer near plastic-to-rubber critical transition produces enhanced elastocaloric effects. *Cell Reports Physical Science.* **3**, 101147 (2022).
6. Marrink, S. J., Risselada, H. J., Yefimov, S., Tieleman, D.P., & de Vries, A.H. The MARTINI forcefield: coarse grained model for biomolecular simulations. *J. Phys. Chem. B* **111**, 7812-7824 (2007).
7. Andrei, A. G. & Fabian, S. Molecular dynamics study on the validity of Miller−Macosko theory for entanglement and crosslink contributions to the elastic modulus of end-linked polymer networks. *Macromolecules* **55**, 8372−8383 (2022).
8. Xia, J., Xiao, Q. & Guo, H. Transferability of a coarse-grained atactic polystyrene model: Thermodynamics and structure. *Polymer* **148**, 284-294 (2018).
9. Nosé, S. A unified formulation of the constant temperature molecular dynamics methods. *J. Chem. Phys.* **81**, 511-519 (1984).
10. Nosé, S. Constant temperature molecular dynamics methods. Prog. *Theor. Phys. Suppl.* **103**, 1-46 (1991).
11. Souza, I. & Martins, J. L. Metric tensor as the dynamical variable for variable-cell-shape molecular dynamics. *Phys. Rev. B* **55**, 8733 (1997).
12. Wall, F. T. Statistic thermodynamics of rubber. *J. Chem. Phys.* **10,** 132-134 (1941).
13. Flory, P. J. & John, R. J. Statistical mechanics of cross - linked polymer networks I. Rubberlike elasticity. *J. Chem. Phys.* **11,** 512 (1943).
14. Yoshida, Y., Yuse, K., Guyomar, D., Capsal, J. F. & Sebald, G. Elastocaloric effect in poly(vinylidene fluoride-trifluoroethylene-chlorotrifluoroethylene) terpolymer. *Appl. Phys. Lett.* **108**, 242904 (2016).

15. Patel, S., Chauhan, A., Vaish, R. & Thomas, P. Elastocaloric and barocaloric effects in polyvinylidene di-fluoride-based polymers. *Appl. Phys. Lett.* **108**, 072903 (2016).
16. Xie, Z. J., Sebald, G. & Guyomar, D. Comparison of direct and indirect measurement of the elastocaloric effect in natural rubber. *Appl. Phys. Lett.* **108**, 041901 (2016).
17. Xie, Z. J., Wei, C., Guyomar, D. & Sebald, G. Validity of Flory's model for describing equilibrium strain-induced crystallization (SIC) and thermal behavior in natural rubber. *Polymer* **103,** 41-45 (2016).
18. Guyomar, D. et al. Elastocaloric modeling of natural rubber. *Appl. Therm. Eng.* **57,** 33-38 (2013).
19. Wang, R. et al. Torsional refrigeration by twisted, coiled, and supercoiled fibers. *Science* **366**, 216-221 (2019).
20. Greibich, F. et al. Elastocaloric heat pump with specific cooling power of 20.9 W g–1 exploiting snap-through instability and strain-induced crystallization. *Nat. Energ.* **6**, 260-267 (2021).
21. Coativy, G. et al. Elastocaloric properties of thermoplastic polyurethane. *Appl. Phys. Lett.* **117,** 193903 (2020).
22. Sagotra, A. K., Chu, D. & Cazorla C. Room-temperature mechanocaloric effects in lithium-based superionic materials. *Nat. Commun.* **9**, 3337 (2018).
23. Sagotra, A. K., Errandonea, D. & Cazorla C. Mechanocaloric effects in superionic thin films from atomistic simulations. *Nat. Commun.* **8**, 963 (2017).
24. Cong, D. et al. Colossal elastocaloric effect in ferroelastic Ni-Mn-Ti alloys. *Phys. Rev. Lett.* **122,** 255703 (2019).
25. Pataky, G. J., Ertekin, E. & Sehitoglu, H. Elastocaloric cooling potential of NiTi, Ni_2FeGa and CoNiAl. *Acta Mater.* **96,** 420-427 (2015).
26. Sebald, G., et al. High-performance polymer-based regenerative elastocaloric cooler. *Appl. Therm. Eng.* **223,** 120016 (2023).
27. Tušek, J. et al. A regenerative elastocaloric heat pump. *Nat. Energ.* 1, 16134 (2016).
28. Bruederlin, F., Ossmer, H., Wendler, F., Miyazaki, S. & Kohl, M. SMA foil-based elastocaloric cooling: from material behavior to device engineering. *J. Phys. D Appl. Phys.* **50**, 424003 (2017).
29. Engelbrecht, K. et al. A regenerative elastocaloric device: experimental results. *J. Phys. D Appl. Phys.* **50**, 424006 (2017).

30. Bachmann, N. et al. Long-term stable compressive elastocaloric cooling system with latent heat transfer. *Commun. Phys.* **4**, 194 (2021).
31. Qian, S. et al. High-performance multimode elastocaloric cooling system. *Science* **380***,* 722-727 (2023).
32. Sebald, G., Xie, Z. & Guyomar, D. Fatigue effect of elastocaloric properties in natural rubber. *Phil. Trans. R. Soc. A* **374,** 20150302 (2016).
33. Maji, P. et al. Strategic fabrication of SEBS composite with high strength and stretchability via incorporation of polymer-grafted cellulose nanofibers for biomedical applications. *Cellulose* **30,** 9465-9484 (2023).
34. Kabirifar, P. et al. Elastocaloric cooling: State-of-the-art and future challenges in designing regenerative elastocaloric devices. *J. Mech. Eng.* **65,** 615–630 (2019).
